# Ultrashort Peptide Self-Assembly: Front-Runners to Transport Drug and Gene Cargos

**DOI:** 10.3389/fbioe.2020.00504

**Published:** 2020-05-29

**Authors:** Seema Gupta, Indu Singh, Ashwani K. Sharma, Pradeep Kumar

**Affiliations:** ^1^Chemistry Department, Acharya Narendra Dev College, University of Delhi, New Delhi, India; ^2^Nucleic Acids Research Laboratory, CSIR-Institute of Genomics and Integrative Biology, New Delhi, India

**Keywords:** amphiphilicity, peptide, self-assembly, drug delivery, tissue engineering

## Abstract

The translational therapies to promote interaction between cell and signal come with stringent eligibility criteria. The chemically defined, hierarchically organized, and simpler yet blessed with robust intermolecular association, the peptides, are privileged to make the cut-off for sensing the cell-signal for biologics delivery and tissue engineering. The signature service and insoluble network formation of the peptide self-assemblies as hydrogels have drawn a spell of research activity among the scientists all around the globe in the past decades. The therapeutic peptide market players are anticipating promising growth opportunities due to the ample technological advancements in this field. The presence of the other organic moieties, enzyme substrates and well-established protecting groups like Fmoc and Boc etc., bring the best of both worlds. Since the large sequences of peptides severely limit the purification and their isolation, this article reviews the account of last 5 years' efforts on novel approaches for formulation and development of single molecule amino acids, ultra-short peptide self-assemblies (di- and tri- peptides only) and their derivatives as drug/gene carriers and tissue-engineering systems.

## Introduction

The global trend is growing toward precise medicines and diagnoses through multi-centered approaches of drug delivery technology. The poor systemic bioavailability, solubility, absorption, and stability of large sized materials pose major challenges in the area of drug delivery. Novel natural biomaterials which can qualify to be biodegradable, biocompatible, non-toxic, renewable, and readily available to deliver therapeutic agents to precise targeted sites in a controlled manner is one of the most sort after research-field. Presently, nanotechnology (Lombardo et al., 2020) has provided a solution by opening up of newer avenues in terms of developing advanced controlled drug delivery and release systems that have met with huge success (Webber et al., 2016; Webber and Langer, 2017; Patra et al., 2018). The customizable nanoparticles with the manipulation in size, surface characteristics and materials used enhance the efficacy of drug delivery in a paramount manner along with the advantage of safer treatment (Eskandari et al., 2017; Rizvi and Saleh, 2018). A relatively newer area to deliver drugs across biological barriers for improved site-specific absorption is also being explored (Kou et al., 2018) i.e., transporter-targeted nanoparticles. A specific application based self-assembled materials, injectable biomaterials, are also being investigated to improve the advancing practices in healthcare (Sahoo et al., 2018b).

**Figure 1:**
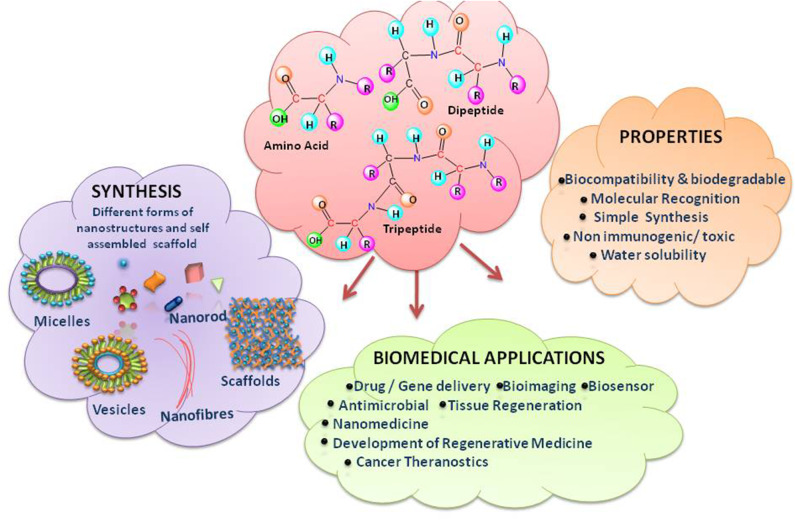
Overview of the projected review.

Uncomplicated to design and synthesize, biocompatible, embedded with appropriate opportunities for chemical alterations, demonstrating molecular selectivity and specific interaction with diverse types of biological systems- all these characteristics make peptides ideal and flexible candidates for constructing tuneable nanostructures with normal end functionalization (Sun L. et al., 2016; Yu et al., 2016; Galdiero and Gomes, 2017) (Figure 1). It is hypothesized that simple amino acids may be the first catalysts for formation of peptide bonds (Luisi, 2015). The evolutionary model put forward by Carny and Gazit on the mechanism of origin of life links the aptitude of short peptides to the creation of the present living systems (Carny and Gazit, 2005). According to this model, the properties like encapsulation, catalytic potential in chemical reactions and a highly ordered template for the assembly of nucleotides, which might be the early events, could lead to create the biological systems. Self-assembling peptides, a category of peptides, assemble spontaneously into ordered nanostructures. Peptides, even as short as dipeptides, are blessed to hold all the desirable molecular information to form well-ordered structures, when worked for nano-scale. The self-assembly of short peptides turning to β-sheet amyloid conformers brings extraordinary structural stability and multi-functionality like; self-replication, catalytic activities and information transfer, which is impossible for the corresponding non-aggregated peptides. The emergence and evolvement of such mutualistic networks may eventually lead to origin of life (Maury, 2018).

Peptide self-assemblies are the gait of 20 amino acids which can be manipulated in terms of number, type, sequence, and side chain groups. These nanostructures can be customized by incorporating modified amino acids in the peptide design to have superior assembling properties and enzymatic stability. This bottom-up science, inspired by the wonders of nature operating on the nanoscale, generates many biological nanostructures such as proteins and DNA/RNA, enabling functioning of life. Even the top-down method, with an advantage over the bottom-up approach supports to discover new peptide sequences aimed to a specific binding site on bio-macromolecules based on their structural properties. It is compelling the scientists to reach after the chemistry of these macromolecules and synthesize newer molecular self-assemblies. The peptide synthesis is a simple and affordable synthetic chemistry via conventional procedures, solution or solid phase. The cost of making peptide is found to be associated with the motif length, purity, chirality of amino acids as well as the expertise of the fabricator. The ability of natural and synthetic amino acid building blocks, both, to form ordered assemblies through self-association process with defined architectures and prominent physical properties has been reviewed by Chakraborty and Gazit (2018). The review by Lee et al., has summarized the building blocks of peptide self-assemblies categorized by their constituting amino acids, the bound chains/motifs, characteristics and regulatory factors (Lee et al., 2019). The study of ordered nanofibres from protein self-assembly in natural systems has been recognized as reductionist approach, wherein, core peptide building blocks could be derived from the parent protein (Guterman et al., 2016).

### Regulators of Self-Assembly

The fundamental mechanisms for the self-assembled nanostructures of different types and structures of peptides are explored regularly (Mandal et al., 2014; Chen et al., 2016; Liao et al., 2016; Fan et al., 2017; Pandit et al., 2018; Mason and Buell, 2019). The constituent amino acid residues govern the supramolecular nanostructures and peptide secondary structures to adopt the conformations led by intra-/intermolecular interactions during peptide self-assembly. The multifunctional materials with highly ordered structures could be achieved by hierarchical architectures with anisotropy and suggested to be facilitated by rational self-control of weak intermolecular interactions regulating the self-assembly process at different steps (Yuan et al., 2019).

### Inter/Intramolecular Non-Covalent Interactions

The design and nature of these ordered nanostructures is the outcome of the synergistic effect of various intermolecular non-covalent interactions, comprising H-bonding, π-π bonding, electrostatic forces, hydrophobic, and van der Waals' interactions. Molecular self-assembly, a more stable structure, is formed under thermodynamic equilibrium conditions by arrangements through various non-covalent weak interactions which can generate assemblies of excipient patterns (Mateescu et al., 2015). This alliance of non-covalent interactions strategizes the self-assembly process and determines the thermodynamic stability, though, kinetic parameters also play a critical factor in concluding the dynamic material (Wang et al., 2016a; Liu et al., 2017).

The hydrogen bonds have the competence to assist the growth of biomolecules in one direction with a long-range order to manifest one-dimensional (1D) nanostructures. The hydrogen bond stacking in case of cyclic peptides has been reported to provide the primary structure and comprehensive cylindrical morphology to the self-assembly (Rho et al., 2019). Besides the contribution from individual amino acids, the peptide backbone itself also provides significant stability through hydrogen bonds (Leite et al., 2015).

Electrostatic interactions portray remarkable performance in the self-assembly of peptides and stabilize the nanostructures. Electrostatic attraction between the positively charged peptide fibril and negatively charged small molecule drugs for controlled drug delivery (Mauri et al., 2017).

Amino acids based on amino acid residues can be categorized into hydrophobic and hydrophilic ones. Peptides can form highly ordered self-assembled superstructures due to their hydrophobic property. These hydrophobic interactions are observed to progress from open networks of secondary structures toward closed cylindrical nanostructures (β-sheets or random coils) (Fu et al., 2014).

van der Waals forces, contemplating entire intermolecular forces and relatively weaker to covalent bonds, play an integral role in supramolecular organization in nanotechnology. van der Waals interactions in plane are observed to control not only the molecular self-assembly structure but their phase transition as well (Gao et al., 2015). The dipole-dipole interactions also support in the self-assembly process and are reported to enhance the mechanical properties as dual non-covalent bonding strategy (Cao et al., 2019).

### Ionic Interactions

The arrays of ionic interactions are also part of the driving energy of the self-assemblies in water along with the hydrophobic interactions and peptide-backbone hydrogen bonds. Self-assembling, ionic-complementary peptides are also being studied by researchers (Chen, 2005). The synergistic effect of both non-covalent and ionic interactions has been reported to provide better stability to the studied hydrogel (Xie et al., 2016).

### Aromatic Interactions

π-π stacking between large π-conjugated surfaces provides an overall stability to supramolecular polymers bound together by non-covalent interactions (Cockroft et al., 2005). Various collagen like peptides mimic the fibril formation and assemble into higher order hierarchical structures through π-π stacking interactions (Chen and Zou, 2019). The hypothesis put forward by Gazit et al. regarding the lead role played by aromatic interactions in the self-assembly of peptide nanotubes/amyloid-like structures has been established time and again (Reches and Gazit, 2005). The aromatic-aromatic interactions have been reported to transform into β-sheet conformation from α-helix on being connected to an aromatic motif at C-terminal (Li J. et al., 2017). Though the importance of aromatic interactions in amyloid formation has been challenged (Lakshmanan et al., 2013) but, are significant in amyloid β-peptide (Genji et al., 2017).

In peptide self-assembly, nonpolar amino acids (aromatic and aliphatic amino acids) aggregate through π-π stacking and hydrophobic interactions, while the polar amino acids, depending on whether they have uncharged or charged residues, stabilize through either electrostatic interactions or hydrogen bonding. The weak bond-based injectable hydrogels based on hydrogen bonding, ionic, hydrophobic and π-π stacking interactions and host-guest chemistry have been reviewed by Ding and Wang (2017). These non-covalent interactions act as driving force in designing the gels (Dou and Feng, 2017). In a characteristic assembly, electrostatic repulsions control the nanofiber length. Addition of a covalent bond forming unit, which could conjugate to the peptide sequence by amide bond condensation altered the balance between hydrogen bond formation and compensated the repulsive electrostatic interactions. Thus formation of covalent bond reinforced hydrogen bonds between peptides enabling the fiber elongation, which otherwise energetically is not possible (Sato et al., 2017). The study on reversible covalent chemistry displayed that the difference in self-assembly modes at the non-covalent level could also be reflected at the covalent level (Komáromy et al., 2017).

It is difficult to predict the combo of all these molecular forces after the peptide self-assembly. Extra facts about the interaction of these forces are essential to plan much efficient, chemically stable peptide self-assemblies.

### Secondary Structural Conformations

Most of the self-assembling peptides are supposed to be readily soluble in water due to the presence of amino acid molecules containing charged residues (alternating hydrophilic and hydrophobic regions), periodically repeated and discrete polar and non-polar surfaces. α-amino acids comprising the peptides have the inclination to adopt various secondary structural conformations like α-helices (Boyle, 2018), β-sheets (Leite et al., 2015), β-hairpins (Nagarkar et al., 2008) and even the folds (Yoo and Lee, 2017; Kulkarni et al., 2019) and this dynamic behavior of the self-assembling peptides at the molecular structural level influences the peptide-based self-assembly processes in water (Gopalan et al., 2015; Bera and Gazit, 2019; Kulkarni et al., 2019). The process continues and these structures assemble further spontaneously to form nanofibers which consequently aggregate into supramolecular scaffolds and can entrap large volumes of water. The hierarchical self-assembly process can potentially stabilize diverse β-sheet hydrogen bonded architectures. Among the secondary structures, hydrogen bonding between carbonyl oxygen and the amino group of every third residue in the helical turn stabilizes an α-helix (each helical turn consisting of 3.6 amino acid residues) while β-sheets originate with hydrogen bonding between two or more β-strands (along which, the backbone of the peptide stretches) (Kulkarni et al., 2019). The self-association phenomenon of aromatic side-chains in β-peptide oligomers supports the helical secondary structure formed by intramolecular backbone-side chain CH-π interactions and yields large vesicles due to the gain in the hydrophobic area (Mándity et al., 2014). In a study, Sarkar et al. (2015) have also demonstrated the effect of solvent interactions on the folding pattern resulting in a change in initial helical conformation and structural diversity of short aromatic γ-peptides. Another architecture, coiled-coil peptide self-assembly, though suffering from the drawback of having longer amino acid sequences compared to other self-assembling peptide systems such as β-sheet fibrillizing peptides or peptide amphiphiles has been reported to offer unique advantages as reviewed by Wu and Collier (2017). All these functional architectures are the result of molecular recognition process and self-assembly led by non-covalent interactions. Zhou et al. enlightened on the adoption of amino acid conformations in the complex interactive process in relation to peptide sequence. The study evidenced that peptides with high sequence similarity could self-assemble into diverse nanostructures though could acquire similar secondary structures while completely different sequences assembled into one type of nanostructures (Zhou et al., 2019).

Various molecular forces make peptides self-assemble in different supramolecular peptides. As per the observations, a peptide with an electrostatically charged/hydrophilic head and a hydrophobic tail would self-assemble in spherical micelles or vesicles which on elongation could lead the way into fibers or tubes, respectively. Peptides with a β-sheet show inclination to assemble into flat structures suchlike tapes or ribbons. Nonetheless, on increasing the concentration of the peptides, these tapes and ribbons could stack on one another and turn into more firmly packed fibers.

A relatively new innovation, the use of co-assembly, to produce nanostructures is also being explored. When an individual component is incompetent to have the basic properties required for the self-assembly, a co-assembly option provides the necessary support. The cooperativity movement is directed by non-covalent interactions, in particular, electrostatic. The combination of experimental justification and computational simulations provides a basic support to identify structural and functional components (Raymond and Nilsson, 2018).

### Amphiphilic Peptides

It is an aqueous peptide self-assembly, characteristically spurred by the presence of amphiphilic character in the monomer units, contains hydrophilic and hydrophobic domains, which impulsively arrange to shield hydrophobic groups and minimize contact with bulk water. These molecules contain one or more alkyl chain tails along with a terminal peptidic head group. The fine tuning of balance among the hydrophilic block with polar amino acids and hydrophobic blocks could stabilize various supramolecular structures by hydrophobic, electrostatic, β-sheet hydrogen bonds and π-π stacking interactions (Mikhalevich et al., 2017; Qiu et al., 2018). A naïve investigation by Accardo et al. (2013) wherein use of an intrinsically disordered peptide as a polar head connected to alkyl chain led to some disorder-to-order transition upon their self-assembly in supramolecular aggregates. The group recommended this kind of ordered core and a “disordered” surface as a potential scaffold for future biomaterials. Furthermore, Tesauro et al. (2019) discussed the versatility of arrangements in side chains, option to load charges/functional groups and select physical and chemical patterns to acquire desired biostructures during the design of peptide amphiphile (PA) and suggested the option of using conformational preferences in structured and/or disordered peptides in the review.

The review by Cui et al. has emphasized on controlling the environment of PA self-assembly manufacturing and details of their applications (Cui et al., 2010). Addition of an ionizable/charged amino acid to the PA structure is suggestive to increase the number of charges per aggregate. A self-assembly model of PAs produced by a C_16_ alkyl tail linked to a chain of two lysines (C_16_K_2_) or three lysines (C_16_K_3_) stand reasonably accurate on the predicted behavior in terms of morphology, size and the state of protonation of the aggregates. The work by Zaldivar et al. revealed that the system followed a charge regulation mechanism and found to decrease electrostatic repulsions between charged lysines (Zaldivar et al., 2019). An atomic level study by Rad-Malekshahi et al. (2015a) analyzed the vesicle surface structure and dynamics of self-assembled nanovesicles along with the intermolecular forces between amphiphilic peptides to improve and tune the biophysical properties of the nanocarrier.

### Impact of Chemical Modifications

Different chemical reactions are employed to construct self-assembled nanostructures (Rasale and Das, 2015). A general approach followed by peptide chemists is to incorporate modifications in the form of easy and reversible cleavable appropriate moieties/groups at the carbonyl–nitrogen bond (readily cleavable urethanes containing appropriate alkyl groups such as benzyl and *tert*-butyl liberating the amino groups), incorporating alkyl spacers of various lengths to modulate the chirality (Panda et al., 2019), π-clamping to tune the reactivity (Zhang C. et al., 2016), backbone amide modifications for peptidomimetic hydrogelators with enhanced stability and mechanical properties compared to peptide hydrogelators (Basavalingappa et al., 2019), enforcing a conformational constraint to prevent β-sheet structure (Bowerman and Nilsson, 2010), formulated sequence patterns to optimize charge distribution for conjugating bioactive cargo (Zhang H. et al., 2017), alternating d/l-chirality for 1D- to 2D-self-assembly (Insua and Montenegro, 2019), a racemic mixture of the mirror-image peptides to provide more rigidity to the gel (Nagy-Smith et al., 2017), alternative hydrophobic and hydrophilic residues for catalytic activity (Song et al., 2018), coordination with metal ions to inhibit amyloid-like structure (Ji et al., 2019), fine-tune assembly for hydrogel (Loic, 2017) and so on. N-Acetylation (compound attached to the amino; N-terminus) and C-amidation (compound attached to the carboxyl; C-terminus) is the most usual policy to stabilize nearly all categories of peptides. The peptides, without modification, perhaps may not be toxic, but the impact after the modification, though also unsettled, is being explored (Soleymani-Goloujeh et al., 2018). Amino acids and short peptides bearing moieties like 9-fluorenylmethoxycarbonyl (Fmoc) (Tao et al., 2016), (Chakraborty and Gazit, 2018), aromatic naphthalene-2-methoxycarbonyl (Nmoc) (Rasale et al., 2015), 9-anthracenemethoxycarbonyl (Amoc) (Gavel et al., 2018a), at N-terminal provide extra advantage to the fabrication of self-assemblies due to inherent hydrophobicity and aromaticity and are also, significant in gel formation (Orbach et al., 2012; Fleming et al., 2013; Singh et al., 2015). *tert*-butyloxycarbonyl (Boc) is also used to protect α-amino group in peptide synthesis (Ragnarsson and Grehn, 2013). Introduction of non-natural d-amino acid at the N-terminus, has shown unexpected effects on peptide secondary conformation and even the biological performance and so is another useful strategy to confer self-assembling properties (Melchionna et al., 2016). Small chemical modifications in peptides at the N- or C-terminus, intrinsic to self-assembly into ordered supramolecular architectures and their biomedical applications, have been reviewed by Rad-Malekshahi et al. (2015b). N- and C-terminal of the aromatic components as well as linker segment and peptide sequence have also been demonstrated to control the self-assembly of aromatic peptide amphiphiles (Fleming and Ulijn, 2014).

Recently, guiding principles to customize the kinetics and morphological changes in supramolecular peptide nanostructures have been introduced by Son et al. (2019) upon exposure of matrix metalloproteinase. These guiding principles enumerate systematic customization of enzyme-responsive peptide nanostructures by exchange of just a few amino acids, for general use in performance optimization of enzyme-responsive materials (Son et al., 2019). The advantages of enzyme-instructed self-assembly (EISA) in triggering the molecular self-assembly *in situ*, by overexpression, to prepare supramolecular biofunctional materials and hydrogels has been reviewed by Gao et al. (2019).

The focus has also turned to design self-assemblies of peptide-based conjugates. The multidisciplinary studies involving conjugated short peptides and single amino acids highlighted the role of conjugated material and broadened the horizon of self-assembling materials (Acar et al., 2017; Edwards-Gayle and Hamley, 2017). The reformed attempts to enhance *in vivo* half-life time and widespread applications of peptides are being made by conjugating them with nanoparticles (Jeong et al., 2018; Spicer et al., 2018; Wang et al., 2018; Jiang et al., 2019). A critical comparison of peptide materials with non-peptide materials has been attempted by Santis and Readnov to count on the contribution of peptide self-assemblies in real-life applications i.e., commercial products (De Santis and Ryadnov, 2015). The use of sequence-specific peptides as biological recognition elements has nicely been reviewed by Slocik and Naik (Slocik and Naik, 2017). The insertion of suitable spacers (charged or neutral) between the hydrophobic region and the peptide are reported to uphold flexibility, mobility, and sometimes increase the solubility of the molecule. The linker is supportive for creating functionalized nanofibrils and expand the modules of chemoselective bio-conjugation approaches in site-specific titivation of self-assembling peptides (Biscaglia et al., 2016; Scelsi et al., 2019). Cui and coworkers (Cui and Chen, 2017) worked on a themed issue envisioned to bring leading researchers working on peptides and peptide conjugates to assess the recent progress in utilizing peptide-based constructs and describe the challenges to interface with biology for specific biomedical applications.

The peptide-templated noble metal catalysts also play an important role in chemical biology (Wang W. et al., 2017). Metal coordination to natural and non-natural binding sites of different peptides has been reported to stimulate the peptide self-assembly (Zou et al., 2015). This knowledge of the forces to obtain an ordered organization can assist innovative peptide based materials for more assorted applications.

### Applications of Peptide Self-Assemblies

#### Peptide Self- Assemblies as Drug Carriers

The rich chemistry of various non-covalent interactions has led to swift development of self-assemblies as drug carriers, particularly, in short peptides (Huang et al., 2013; Panda and Chauhan, 2014; Iglesias and Marchesan, 2017; Amit et al., 2018; Raza et al., 2018; Mishra and Jyoti Panda, 2019).

**Figure 2:**
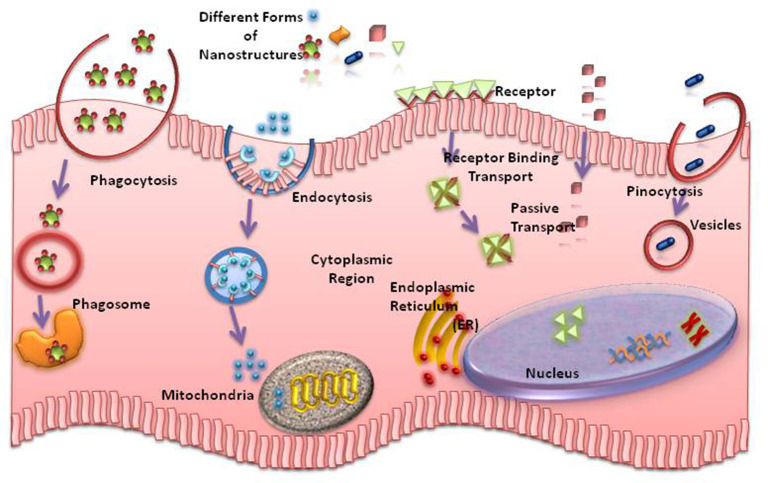
Different routes of entry of nanostructures into the cells.

The impact of finite peptide nanostructures for the development of systemic therapeutic delivery vehicles is, in particular, of interest, as the length of the assembly plays important roles during cell uptake and tissue penetration (Mendes et al., 2013) (Figure 2). A strategy has been reported, where the length of charged peptide-amphiphile supramolecular assemblies could be controlled through covalent bond formation (Sato et al., 2017).

The extensive non-covalent interactions provide several advantages in developing self-assembled materials for drug-delivery (Leite et al., 2015). These are mainly at the three working stages, namely, drug loading, self-assembly–drug conjugate transport, and finally the cellular drug delivery (Doane and Burda, 2012; Habibi et al., 2016; Fan et al., 2017). Devadasu et al. have suggested that understanding of the drug and disease is of utmost importance before designing a delivery system (Devadasu et al., 2012). The self-assembled-ordered structures of peptides with extensive π-π and hydrogen bonding, also a precondition for semiconductor properties, has aspired the scientists to assemble such structures for biological semiconductors along-with biocompatible and drug release materials (Tao et al., 2017). A review by Sis and Webber has discussed about the basic designs in peptide self-assemblies and ways to mend the efficacy of drug delivery (Sis and Webber, 2019). Self-assembled photosensitizers resulting from amphiphilic dipeptide- or amino-acid-tuned for photodynamic therapy (PDT) have been reported (Liu et al., 2016). The tuneable size, surface charge and multi-responsiveness toward pH, detergents, and enzymes suggest the simple and efficient self-assembled method to deliver photosensitizers (Li L.L. et al., 2018).

Amphiphilic peptides have brought a paradigm shift toward self-assembled PA in drug delivery. The amphiphilic peptides provide a lot of option in both linear and cyclic peptide sequences, side chains loaded with charges, self–assembled as vesicles, micelles, nanofibers and nanotubes for delivery systems and for other biotechnological applications (Goel et al., 2015). An account on the versatility of cyclic peptides and the safety measures in terms of size control, length and bundle width of nanotubes during the successful delivery of active pharmaceutical ingredients has been presented in a mini-review by Hsieh and Liaw (2019). The impact of amino-acid side-chains or covalently linked hydrophobic chain in PA on their stimuli-responsive drug delivery applications has been reviewed by Song et al. (2017).

Another strategy is to use the self-assembly of amphiphilic drug molecules to do the drug-loading and then deliver the cargo as well on its own. In case of low water-solubility of the drug, hydrophilic segments may be conjugated to bestow amphiphilic behavior. The conjugation pushes the peptide sequences for one-dimensional elongation through β-sheet formation. Their architecture fundamentals administer the self-assembly of PA's into supramolecular systems to be applied in drug delivery (Lock et al., 2013).

Ample attention is also being directed to cell-penetrating peptides (CPPs), as carriers for intracellular transport cargoes such as siRNA, nucleic acids, proteins, various nano-particulate pharmaceutical carriers (e.g., liposomes, micelles), small molecule therapeutic agents as well as quantum dots and MRI contrast agents (Brasseur and Divita, 2010; Bechara and Sagan, 2013; Choi and David, 2014; Copolovici et al., 2014; Wang et al., 2014; Huang et al., 2015; Skotland et al., 2015; Dinca et al., 2016; Guo Z. et al., 2016; Kurrikoff et al., 2016; Lehto et al., 2016; Guidotti et al., 2017; Hoffmann et al., 2018; Panigrahi et al., 2018; Ramaker et al., 2018; Vánová et al., 2019), since these are internalized by cells in an exceedingly effective manner. The study by Ramaker et al. observed a statistically substantial dependence of CPPs' uptake efficiency on both net charge and peptide length; longer CPP possibly due to more ordered α-helical structure with high charge could ferry conjugated cargo across membranes more efficiently (Ramaker et al., 2018). The non-covalent approach for complexing CPPs to nucleic acids or viruses has shown better gene delivery both *in vitro* and *in vivo* (Alhakamy et al., 2013). Gallo et al. have provided a comprehensive list of recognized CPPs along with their reported applications (Gallo et al., 2019). Another review by Borrelli et al. has discussed biological properties of CPP upon conjugation with specific molecules with special emphasis on uses in cancer therapy (Borrelli et al., 2018).

Nanomedicines directly assembled from pharmaceutical ingredients, termed as small molecule nanomedicines (SMNs), have the potential to improve the drug delivery efficiency, biosafety and largely reduce the research and development cost. Xue et al. (2020) highlighted the recent advances in a section on drugs and photosensitizers with peptides and exhibited advantages of SMNs in the review article.

Tailored drug delivery vehicles are continuously gathering attention. Peptide–drug conjugates (PDCs) enable selective delivery of cytotoxic cargoes to target cells (Ma et al., 2017; Wang et al., 2017a; Wang W. et al., 2017; Vrettos et al., 2018). PDCs have exclusive and precise features to build one-component nanomedicines (OCNs) containing only one type of chemical substance. These OCNs do not require additional carriers. In fact, these are equipped with desired physicochemical features to involuntarily aggregate as well as accumulate at target sites (Su et al., 2015). Since peptides can be manufactured effortlessly in large quantities and need simple purification, their range of selection of peptide sequences as per the requisite physicochemical properties like stability, solubility, overall charge and availability of the characteristic groups for the conjugation with the therapeutic payload, these are considered as sought-after prodrugs (He et al., 2019).

Peptide-based hydrogels is another class of drug delivery vehicles programmed via drug encapsulation or conjugated covalently with therapeutics. Small peptide molecules, in general, have the desired state to form specific secondary structures in solution and then self-assemble into fibrillary network under various physical conditions (Fu et al., 2013; Tomasini and Castellucci, 2013; Zhang L. et al., 2015). An analysis of the structural/molecular features of different β-sheet peptide hydrogels and their relation to mechanical properties to design effective hydrogels has been reviewed by Rodriguez et al. (2016). The sticky-ended fibrillation designs, applied in DNA and coiled fibers, have inspired Sarkar et al. (2014) to develop a strategy to form staggered triple helical species assisted by interchain charged pairs. The comparison between the two classes of collagen mimetic peptides having same composition but different domain arrangements showed that the larger nucleation domains resulted in rapid fiber formation and gelation while short nucleation domains left the peptide soluble for longer period (Sarkar et al., 2014). The thixotropic supramolecular hydrogels formed in response to external environmental stimuli have wide ranges of potential biological applications (Levin et al., 2014; Seow and Hauser, 2014; Loic, 2017; Zanna and Tomasini, 2017; Mondal et al., 2020). The stability of peptide-based hydrogels, specially to enzymatic degradation has been reviewed by Yadav et al. (2020). The composition of hydrogel material with tuneable properties controls the release of sensitive drugs (Du et al., 2015; Raza et al., 2018). Various methods to design and control the self-assembly mechanism in hydrogels for drug-delivery have been elaborated in the literature (Altunbas and Pochan, 2011; Briuglia et al., 2014; Yu et al., 2015; Li et al., 2019b). A review by Zhang et al. has highlighted on tuning the unique features/morphology of the nanostructures functions formed during the gelation process by controlling the morphology-dependent variations (Zhang L. et al., 2015). The loaded drugs not only get physically trapped, but also bring morphological modifications in the peptide hydrogel (Kurbasic et al., 2017; Parisi et al., 2019), thus converting drug–peptide co-assembly into a supramolecular hydrogel. The attempts have also been made to design peptide-based supramolecular hydrogels for protein drug delivery and gene therapy (Li Y. et al., 2016; Youngblood et al., 2018). The incorporation of non-viral vectors within hydrogels to promote tissue regeneration is another challenging area (Rivas et al., 2019). A photo-cross-linking strategy, based on the ruthenium-complex-catalyzed conversion of tyrosine to dityrosine, to enhance the mechanical stability of nanofibers by 104-fold with a storage modulus of ~100 kPa (perhaps, one of the highest reported so far among the small peptide hydrogels), with potential to be used in tissue engineering and controlled drug release has been reported by Ding et al. (2013). The low molecular mass organic gelators (LMOGs) have gained much interest in recent years with impending applications in drug delivery and tissue engineering (Sagiri et al., 2014; Skilling et al., 2014; Li Z. et al., 2016). The work on nanostructures containing *D*-amino acids, for their role in biologics delivery is also catching attention, but has a long way to go (Wang H. et al., 2016). The covalent conjugation between a drug and a *D*-peptide impacts the gelation properties of a hydrogel including its biostability (Li et al., 2012). Insertion of *D*-amino acids is reported to twist heterochiral self-assembled peptide hydrogels. Its impact on the drug delivery along with other biological performances makes them versatile tools for therapy in future (Fichman and Gazit, 2014; Melchionna et al., 2016). The use of low molecular weight compounds in hydrogels for drug delivery is turning distinctive due to easy injectability, responsiveness to various stimuli and comfort of synthesis (Raeburn et al., 2013; Mayr et al., 2018). Even the presence of a single amino acid can influence the donor–acceptor charge-transfer interaction in a two-component co-assembled nanofibrous hydrogel (Nelli et al., 2017).

β-Hairpin hydrogels, a subgroup of hydrogels, are another exciting candidates as drug delivery vehicle. These are formed through a molecular self-assembly mechanism which occurs only after desired triggering of intramolecular peptide folding. A review by Worthington et al. (Worthington et al., 2017) has discussed the physical properties of this kind of hydrogel network and material properties which can be used for drug delivery.

Another class of self-assembling peptides, multi-domain peptides (MDPs), allows a wide range of modifications in β-sheet motif without disruption so that the materials for delivery can be trapped within the hydrophobic core of the nanofiber depending on the MDP design and cargo. (Kumar et al., 2015; Li I.C. et al., 2016; Moore and Hartgerink, 2017; Lopez-Silva et al., 2018; Chen and Zou, 2019) In a two-component system, in which a porphyrin cap is combined with a cyclic peptide the combination of various binding forces, e.g., hydrogen bonding, metal coordination, and dynamic covalent bonds, allows the delivery of encapsulated ligand (Ozores et al., 2017). These supramolecular injectable biomaterials, that can mimic the natural extracellular matrix nanostructure and show marked cellular infiltration, are ideal scaffolds for tissue engineering strategies. The self-assemble process of MDPs to a nanofibrous hydrogel requires the peptide sequence containing a core of alternating hydrophilic and hydrophobic amino acids and flanked by presence of charged amino acids which further modifies to nanofibers with bilayered β-sheets. These are further modified to a viscoelastic hydrogel processed via nanofiber elongation and cross-linking. The flexible short β-structure and the governing strong forces allow modifications to incorporate functionality, and so are the attractive choices for research (Li and Hartgerink, 2017; Carrejo et al., 2018).

As mentioned earlier, the peptide self-assemblies may have various morphologies and accordingly possess impressive range of applications though sometimes these might be undervalued. To overcome these limitations, assembly of multiple peptidic components can result in a broader range of applications as compared to the self-assemblies of either component. This epitome expands the conformational space of peptide self-assemblies in terms of structural and functional complexities (Makam and Gazit, 2018; Diaferia et al., 2019). For example, mixed dipeptide gelators are assumed to co-assemble to form fibers containing both the gelators randomly. But the pH triggered methodology, determined by the pKa of the gelator, a chemically programmed method, could alter the rate at which self-sorting occurs and forms self-assembled networks (Morris et al., 2013). These sophisticated multicomponent peptide assemblies are being seen as the next-generation bio-inspired materials (Draper and Adams, 2018; Raymond and Nilsson, 2018).

**Figure 3:**
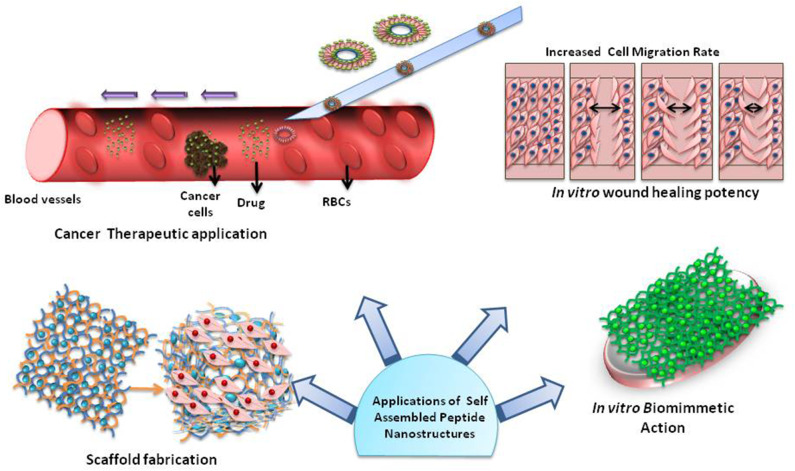
Applications of self-assembled peptide nanostructures.

Tissue engineering is a special branch among self-assemblies whose perseverance is the replacement of damaged tissues with newly engineered tissues to restore the normal activity of the target organ, tissue or system. The rudimentary requirement of tissue engineering is a scaffold to sustain the cells and escort the regeneration of the new tissue (Figure 3). The self-assembling peptides are able to serve this resolute as scaffolds. The special focus in the review articles of hydrogels and porous scaffolds remains in controlled release of drugs from the tissue engineering platforms (Boekhoven and Stupp, 2014; Loo et al., 2015; Rambhia and Ma, 2015; Koutsopoulos, 2016; Banerjee et al., 2018; Lee, 2018; Inaba and Matsuura, 2019). One of the very important areas in tissue engineering is to explore the ways, to modify the properties of the materials, including mechanical and chemical functionality through changes at the sequence level to influence the cell behavior and modulate the scaffold stability (Zhou et al., 2014). Abbas et al. has reviewed the role of peptide and protein self-assembly in photodynamic and photothermal therapy (Abbas et al., 2017). There is a growing interest in the self-assemblies led by non-covalent hydrogels for three-dimensional cell scaffolding applications. The role of ultra-short molecules, namely, dipeptides and amino acids, holds special position in this area (Ryan and Nilsson, 2012). The collaboration between the molecular structure of the assembled materials and the mechanism of self-assembly grips the nerve on evolving biochemical and viscoelastic properties in this network (Maude et al., 2013). In one of the literature reports, Sarkar et al. (2018) have included an elaborated discussion on possible approaches to assimilate the functionality of peptide scaffolds that may be implanted *in vivo* at the site of ischemia for application in functional tissue regeneration. A review article by Rubert Pérez et al. (2015) and highlights the self-assembled peptides through solid-phase peptide methodologies, wherein the accurate amino acid sequence can be selected, for constructing bioactive matrices for regenerative medicine. Major advantages, promising applications and current limitations of peptidic materials has been reviewed by Pugliese and Gelain (2017).

Beyond the biological functions, designing peptides, with desired structures and functions by mimicking natural supramolecular systems, are in the air which can provide innovative designing principles to intricate “molecular robots” as next-generation peptide nanomaterials (Inaba and Matsuura, 2019). Handelman et al. (2016b) studied the reconstructive phase transition exhibited by some of the self-assembled ultrashort di- and tripeptide nanostructures to modify optoelectronic properties followed by the appearance of visible photoluminescence. The physics of light propagation (functional properties of linear and non-linear light propagation) in nanostructures of biological origin has been exploited to develop novel integrated nanophotonic devices (Handelman et al., 2016a).

Though the role of self-assembled peptides in drug and gene delivery is blossoming and there is a substantial flow in the research and review articles as mentioned above, but the short peptides (di- and tri-peptides) appear more promising and game changers, due to their simple structure, cost-effectiveness, non-toxic/non-antigenic nature and superior biocompatibility with enhanced bioactivity (Panda and Chauhan, 2014; Goel et al., 2015; Alam et al., 2016; Guo C. et al., 2016; Habibi et al., 2016; Hamley, 2017; Mishra and Jyoti Panda, 2019; Ni and Zhuo, 2019). Furthermore, the low molecular weight of short peptides allows purification via simple HPLC techniques. This review article has been conceptualized to express the potential of nanostructures resulting from single molecule of amino acids, di- and tri-peptides in the field of targeted biologics delivery and tissue engineering.

### Single or Modified Single Amino Acids

Preparation of nano-shaped aggregates by self-assembly of simple building blocks is fascinating. It is quite startling that a self-assembly of a single amino acid, H-Phe-OH (Adler-Abramovich et al., 2012), was reported in 2012 much after the self-assembly of a dipeptide, H-Phe-Phe-OH (Reches and Gazit, 2003), in 2003. Single amino acid in nanodomain with a stable structure is rare. The chemical modifications of single amino acids have also been investigated for their potential in self-assembly (Chakraborty and Gazit, 2018). The aromatic α-amino acids; H-Phe-OH, H-His-OH, H-Tyr-OH, and H-Trp-OH, have been found to generate ordered self-assembled architects such as fibrils, ribbons, rods, and twisted nanosheets on varying the solvent systems (Singh et al., 2017).

The nanoparticles formed by a single amino acid derivative, a tryptophan derivative, Fmoc-Trp(Boc)-OH, with the molecular weight of 526.6 Da, showed biocompatibility and ability to release the encapsulated bioactive molecules to various cells by Dube et al. (2017). Protection to N-terminus with the Fmoc group and to the side chain with Boc group impacts the nature of peptide self-assembly and overall morphology. The spherical nanoparticles of Fmoc-Trp(Boc)-OH, with hollow interior at pH 6, showed no significant change in structure/morphology with change in pH and retained stability toward thermal perturbations upto 3 months. This asserted the overall impact of Fmoc and Boc groups. Efficient loading and release capabilities along with enhanced toxicity against cancer cells emphasize the role of self-assembly of a simple amino acid as a drug carrier (Tao et al., 2015; Zanna et al., 2015; Dube et al., 2017).

The study to unravel the stimulatory effect of H-Trp-OH on insulin absorption established that H-Trp-OH also possesses bio-enhancing effect as compared to other hydrophobic amino acids, viz., phenylalanine (Phe), proline (Pro) and isoleucine (Ile). The hypoglycemic reaction and surface plasmon resonance (SPR)-based assay showed enhancement in the oral absorption of insulin but without intermolecular interaction. Further, it showed the ability to enhance intestinal absorption of fluorescently labeled hydrophilic dextrans as well as GLP-1 and Exendin-4 peptide drugs (Kamei et al., 2018). H-Arg-OH, as single amino acid, has also shown the potential as insulin absorption enhancer and can be developed as oral delivery systems for insulin (Kamei et al., 2017). Likewise, the hydrophobic and π-π interactions between Fmoc-Lys-OH and pyrrole groups of Chlorin e6 (Ce6), a hydrophobic photosensitive drug, led to the formation of co-assembly, which showed an improved cellular uptake. The results advocate the promising potential of a non-toxic photosensitizer delivery system (Liu et al., 2016).

The pyrene conjugated H-Phe-OH derivative, Pyrene-Phe-OH, displayed its ability to gelify in aqueous solutions over a wide range of pH. The change in pH brought about significant alterations in its properties, namely, (i) a distinct change in morphology (nanoscale) from a chiral (left-handed helical) nano-fibers to achiral (non-helical) tape like nanofibers with increase in pH, and (ii) change in thixotropic (macroscale) property. The thixotropic behavior facilitated the encapsulation of vitamin B_12_ and an anticancer drug, doxorubicin (Dox), within the hydrogel and sustained release paving way to its drug-delivery applicability. The worth mentioning is that Pyrene-Val-OH was unable to form gel owing to absence of π-π interactions for self-association and the gelation process (Nanda et al., 2013).

The hydrophobic interactions hold good position in self-assembly to gel formation. Interestingly, in a research article, it has been shown that phenylketonuria formed due to defective phenylalanine hydroxylase, the H-Phe-OH level in brain increases and the self-assembly forms toxic amyloid fibrils by hydrophobic interactions. The administration of H-*D-*Phe-OH converts the fibrous formation route of H-Phe-OH to flakes formation and the flakes further restrict H-Phe-OH to form fiber. This slows down the toxic fibril formation (Singh V. et al., 2014), though doxycycline is observed to counteract these toxic effects (De Luigi et al., 2015).

The role of aromatic moieties is quite important in the self-assembly of ultrashort peptides, exclusively Fmoc-peptides, to form hydrogels (Orbach et al., 2012). Small angle neutron scattering (SANS) technique has been used to have a view on gel properties and cross-links of the fiber structures of low molecular weight gels formed by dipeptide gelators (Fleming et al., 2014; Mears et al., 2017). The presence of Fmoc protecting group enhances the hydrophobicity of H-Phe-OH, a hydrophobic amino acid, and lowers the solubility in water. The hydrophobicity of phenyl ring has been reported to contribute toward hydrogel formation ring while its aromaticity brings thermal stability to the supramolecular hydrogel system (Murali and Shanmugam, 2019). The study of the recipe of phenylalanine and Fmoc system for gel formation concluded that the covalent linkage between the two is highly imperative to provide accurate configuration and interactions. The methylene side chain serves in stacking process (in buffer conditions) to facilitate gel formation along with the non-covalent ionic interactions and hydrophobic stacking interactions during close proximity of Phe ring and Fmoc moiety in space. The gel showed enhanced dye diffusion and faster gel erosion at higher temperature suggesting further exploration of the molecule in drug delivery (Singh et al., 2015). The unidirectional hydrogen bonding of the carbamate group within Fmoc-Phe-OH assemblies directed the assembly into 1D fibrils while Fmoc-peptoid analogs displayed 2D/3D morphology due to the alteration in H-bonding (Rajbhandary et al., 2018).

**Figure 4:**
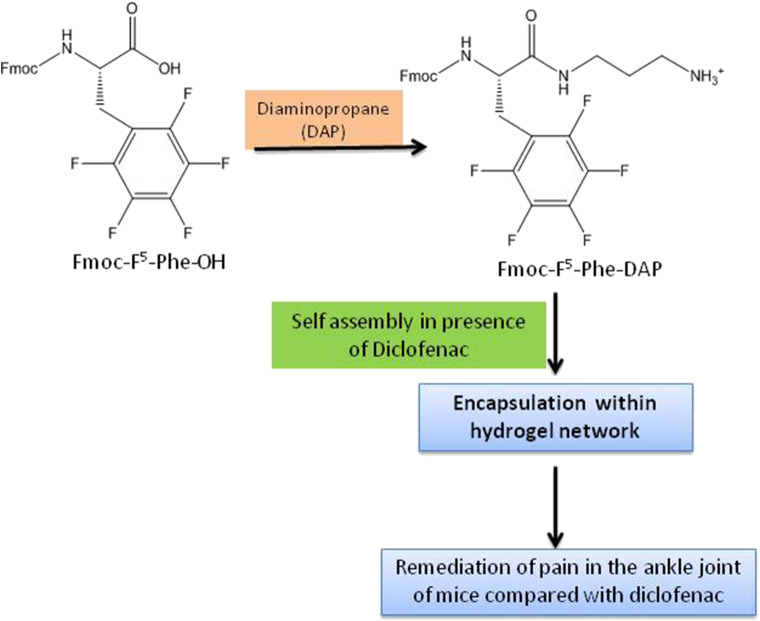
Diclofenac—entrapped gel for pain management.

Fmoc-Phe-OH derivatives, modified at the carboxylic acid position with diaminopropane (DAP), (non-fluorinated Fmoc-Phe-DAP, monofluorinated Fmoc-3F-Phe-DAP, Fmoc-F^5^-Phe-DAP, and co-assembly of Fmoc-Phe-DAP and Fmoc-F^5^-Phe-DAP) twisted to hydrogels on addition of physiologically relevant sodium chloride concentrations. The hydrogels exhibited encapsulation of non-steroidal anti-inflammatory drug, diclofenac, during the self-assembly process and *in vivo* drug release profile showed their usefulness as injectable materials. The release study of diclofenac, which decreased from Fmoc-F^5^-Phe-DAP to monofluorinated to the non-fluorinated gelator, demonstrated the role of the molecular structure of the gelators advocating the importance of aromatic benzyl side chain for π-π interactions between the gelator and cargo (Figure 4) (Raymond et al., 2019).

An amino acid derivative, *N,N*′-dibenzoyl-L-cystine (DBC), has been turned into a supramolecular hydrogel, as the carrier of salicylic acid (SA), by adjusting the pH of the solution. The DBC molecule with two amide groups and two carboxyl groups was able to oblige as hydrogen-bond donors and acceptors, respectively. The self-assembly of DBC molecules was facilitated by strong intermolecular hydrogen bonds linking neighboring amides and carboxylic acid as well as π-π stacking interactions amid aromatic rings, and hydrogen bonds among water molecules. The pH-responsive hydrogel along with thermo-reversibility and suitable mechanical properties is another addition to the controlled drug release system (Zhong et al., 2019).

Not much has been reported about fluorescent peptide nanotubes. Babar and Sarkar (Babar and Sarkar, 2017) synthesized stable, homogeneous nanotube type aggregates from individual amino acids, H-Trp-OH and H-Tyr-OH, retaining the characteristic fluorescence, which can be used as drug carriers. A simple approach, sonication followed by deposition onto brass stub, was used in the synthesis which could be extended to various nanotubes from single or mixture of amino acids. An inexpensive derivative of tyrosine, H-Tyr(t-Bu)-OH, is perhaps one of the lightest reported low molecular weight organogelator and only natural amino acid-derived gelator, decked with free -NH_2_ and -COOH groups. The analysis outcomes made this molecule more special as the gelation could be carried out in all classes of solvents; be it polar, protic, apolar, etc. The gelation in sunflower oil and diesel makes it an appropriate candidate for drug delivery (Aykent et al., 2019). Further investigations revealed the same capability of other enantiomer, H-*D*-Tyr(tBu)-OH to form the organogel while racemic H-*DL*-Tyr(tBu)-OH was unsuccessful to do so. The new work also exposed the role of tert-butyl moiety as H-Tyr-OH, H-Phe-OH and H-Tyr(Me)-OH could not form gel while H-Tyr(tBu)-OH could do so as mentioned above (Aykent et al., 2019).

Injectable self-assemblies of derivatives of H-Ala-OH, turned into hydrogels, were used to attach Dox, through an imine bond and the resulting Dox-Gel showed regression in tumor. The presence of rotationally-flexible, an aromatic N-protecting group on the H-Ala-OH accentuated the nanofiber creation. The intermolecular hydrogen-bonding in the derivatives, formed with the addition of carboxamide and hydrazide units at the carboxylic end of H-Ala-OH, H-Ala-CAM and H-Ala-HYD, respectively, along with the protonated H-Ala-HYD+, aided in the formation of gel. The imine bond between the hydrogel and drug was reversible which could cleave in the surrounding areas of the tumors and release the drug. The proposed work highlighted the use of self-assembly of molecules with molecular weight <300 Da in anticancer therapy (Singh M. et al., 2014).

A metallo-hydrogel was fabricated using H-Val-OH based ligand, L-3-methyl-2-(pyridine-4-ylmethylamino)-butanoic acid and Zn(II). The results revealed reversible gel-to-sol and xerogel-to-gel phase transitions and demonstrated a release of a polar drug stimulated by change in pH (Saha et al., 2014).

A two-component assembly of Fmoc-Tyr-OH and Fmoc-DOPA-OH (DOPA: 3,4-dihydroxyphenylalanine) resulted in a macroscopic structure with different characteristics from formed by modified amino acids hydrogels individually (Fichman et al., 2015). The amino acid H-Phe-OH in its zwitterionic state, under fibrillization conditions, is reported to be stabilized by hydrogen bonds and aromatic interactions network while organized into an ordered β-sheet-like layered assembly (Mossou et al., 2014). All coded amino acids are suggested to display a layer-like assembly (resembling supramolecular β-sheet structures) which is stabilized by α-amine to α-carboxyl H-bonds regardless of the presence of different side-chains that remarkably differ in their chemical properties though these side-chains govern the higher order organization of the layers. Overall, it recommends that the generic inclination of peptides and the proteins backbones to assemble as layered organizations might be ensued from their basic building block, the amino acid (Bera et al., 2018). Co-assembly, though an efficient strategy to form supramolecular structures, suffers in its tailor made functionality due to the lack of knowledge on structural correlation amid different amino acids. This makes the prediction on the resultant co-assemblies a difficult job. Bera et al. have demonstrated that the co-assembly of naturally occurring amino acids with similar chirality is strongly steered by their interlayer separation distances. a basic rule to predict the supramolecular co-assembly relationship with structure (Bera et al., 2019).

The role of disubstituted 1,2,3-triazoles, with the ability to mimic a trans- or a cis- configuration of the amide bond, prompted the researchers to use it as a co-assembly of isosteric amino acid-based hydrogelators. An amphiphilic N-stearoyl-L-glutamic acid (C18-Glu) and its analog with amide moiety replaced by 1,4-disubstituted 1,2,3-triazole unit (click-Glu) displayed distinctive nanostructures due to different hydrogen-bonding configurations. In C18-Glu, the polar protic environment (with intermolecular hydrogen-bonding between the amide NH-bond and the CO-group of the acid moiety next to the chiral center) favored nano-almond crunch-like structures while the non-polar environment preferred (with equal contribution from both intermolecular and intramolecular hydrogen-bonding) formation of nanofibers. The reverse was true for its analog click-Glu. The co-assembly, triggered by the formation of anticipated nanostructures in specific solvents, was applied for the release of an antibiotic, vancomycin (Bachl et al., 2015).

### Dipeptides for Biologics Delivery

Since the beginning of the journey of the self-assembly, H-Phe-Phe-OH, the dipeptide, reported for the first time by Gazit and his group (Reches and Gazit, 2003), has taken the center-stage responding to its various roles. The core-recognition motif of the Alzheimer's disease associated β-amyloid polypeptide, has potential hydrophobic and hydrophilic moieties which are key parameters for molecular assembly. Other than self-assembly by itself, H-Phe-Phe-OH also functions as a co-assembling peptide for adjusting the self-assembly of various functional molecules, providing an easy and efficient method to modulate the morphology and property of these functional molecules. The potential applications of self-assembled dipeptides are catching up in the field of drug delivery and tissue engineering, as these moieties frame into variety of nanostructures, such as, spherical vesicles, nanotubes, and nanowires. The modes of interaction in general are π-π stackings, hydrophobic associations, electrostatic interactions, and hydrogen-bonding. Various morphologies based on free and protected H-Phe-Phe-OH molecule (Ribeiro et al., 2019) and its analogs/derivatives forming fibrils (Reches and Gazit, 2005), vesicles (Guo et al., 2012), tubes (Reches and Gazit, 2006), wires (Huang et al., 2014; Marchesan et al., 2015b), plates (Tamamis et al., 2009), sheets, flakes (Singh V. et al., 2014), necklaces (Yuran et al., 2012) could be demonstrated by manipulating the stimuli and physical/chemical conditions (Adler-Abramovich and Gazit, 2014; Datta et al., 2018, 2019). Concentration dependent ordered nanoarchitectures such as planar bilayers and other diverse shapes of vesicles, namely, discoid, toroid, ellipsoid, and pot-shaped, have been reported by Guo et al. (2012). Further, by chiral control at different stages, two elementary forms of the peptide self-assembly for the helical twisting of the β sheets were developed. These could turn into wide variety of hierarchical chiral nanostructures, viz., tube-like helical ribbons, twisted ribbons, big twists, nanoscrews and nanosprings (Wang et al., 2015). Even the rarely observed toroid nanostructures could be seen in H-Phe-Phe-OH and H-Phe-Phe-Phe-OH co-assembly (Guo C. et al., 2016) and triaromatic system, Z-Phe-Phe-OH (Z= benzyloxycarbonyl) (Brown et al., 2018).

In one of the studies by Wang et al. (2016b) involving H-Phe-Phe-OH dipeptide molecules, it has also been reported that the non-covalent interactions between trace solvents and peptides performing as solvent-bridged hydrogen bonding lead to directional hydrogen bonding between C-O and N–H in these molecules without inducing π-π stacking. It can promote long-range-ordered arrangement to form nanofibers /nanobelts preferentially along one dimension. The study on H-Gly-Pro-OH dipeptide has supported the ability of n → π^*^ interactions between carbonyl groups for the stability of the structure (León et al., 2019). The nanotubes formation in self-assembly of a dipeptide, (S,S)-3-amino-2-(2-fluorophenyl)-3-phenylpropanoic acid and H-Ala-OH, has been observed to be supported by intermolecular N–H···O hydrogen bonds, Cπ-H···O, Cπ-H···F, and van der Waals interactions but no evidence of the presence of stacking interactions between the phenyl moieties has been observed by Bonetti et al. (2015).

The ability of peptides, even the ultrashort peptides as small as dipeptide, to form nano- and microstructures born with unique physical properties is the product of reductionist approach. It proposes the creation of well-ordered, amyloid-like β-sheet-rich assemblies comparable to supramolecular structures made of much larger proteins. This approach is exploited for the design and synthesis of peptide structures of technological utilization while establishing simple *in vitro* model systems to study the parent architecture. This has also resulted in the developing new bio-inspired configurations that could mimic the naturally-occurring architectures while keeping their functional properties intact, or even sometimes new functionalities are born. The similarity in the assembly mechanism between H-Phe-Phe-OH nanostructures and the aromatic amino acid containing amyloid fibrils inspired Brahmachari et al. to use the reductionist approach. Herein, a screening model was set up to identify molecules possibly capable of interfering with the aggregation process and their mode of action on the modulation of both the assembly and disassembly processes of H-Phe-Phe-OH assemblies (Brahmachari et al., 2017). Gazit is pioneer to demonstrate ultrashort peptides forming ordered assemblies by reductionist approach. His review on the reductionist approach educates on future of minimalistic peptide structures and the latitude of bioinspired self-assembly by the final reduction from very short peptides to extremely small metabolites (Gazit, 2018).

The analysis on temperature dependence of the kinetics of H-Phe-Phe-OH assembly, within the skeleton of crystallization theories, exposed that the transition state from solution to crystalline aggregates is enthalpically unfavorable-entropically favorable, qualitatively similar to longer sequences (Mason et al., 2017). Hydrophobic dipeptides, based on crystallization mode, could be categorized as self-assembled crystals composed of (i) elongated helical tubes; narrow hydrophobic channels and (ii) compact helical tubes; wide hydrophilic channels. The molecular mechanism build on density functional theory, advised by González-Díaz et al. recommends the position of the side chain, where it branches, during crystallization, to be the determining factor to drive the dipeptides into either hydrophobic or hydrophilic channels (González-Díaz et al., 2019). The structural transformation of a dipeptide self-assembly just by changing the types/ratios of the metal ion or the dipeptide to inhibit amyloid-like structure has been presented by Ji et al. (2019).

In a pair of dipeptide enantiomers, it is natural for both to exhibit opposite circular dichroism and handedness in self-assembly. In case, one of the amino acids is chiral, this chiral one exploits handedness. The situation is ambiguous, if both the amino acids are chiral. Fu et al. selected four dipeptides derived from *L*- and *D*-alanines and found that the chirality of the alanines at the terminals controls the handedness of their self-assemblies (Fu et al., 2013). The molecular hydrogelators, made of *D*-amino acids, have been shown to improve the selectivity of non-steroidal anti-inflammatory drugs (NSAIDs) (Li et al., 2012). It suggests that the chirality of the C-terminal amino acid commands the chiral orientation of the supramolecular helical nanostructures. In the study of dipeptides, viz., H-Phe-Phe-OH/H-Ala-Ala-OH, with NSAIDs, self-assembled as hydrogels, the peptides made of *D*-amino acids provided assistance to preserve the actions of NSAIDs (Li et al., 2013). Erdogan et al. (2015) observed morphological differences in H-Ala-Val-OH and H-Val-Ala-OH dipeptide molecules in the same solvent medium, though particular solvent property having impact on morphology difference could not be fixed. The two peptides differ in terms of the positional disorder of methyl group side chains in L-Val residues and torsion angles. The symmetrical intra- and intermolecular H-bonds, missing in H-Ala-Val-OH, but, present in H-Val-Ala-OH dipeptides were suggested to be accountable for long-range-ordered structures. The position and as well as number of methyl groups at the α carbons on N-terminus and C-terminus Fmoc-Phe(CH_3_)-Phe-OH or Fmoc-Phe-Phe(CH_3_)-OH has been observed to impact supramolecular nanostructure and the ability to form hydrogel (Arakawa et al., 2020).

An article dedicated to the role of dipeptides in diverse fields has been published by Panda and Mishra (2016). The bioinspired dipeptides in innovative emerging field of photoelectronics have been highlighted by Chen C. et al. (2015). Marchesan et al. discussed the preparation, characterization and pointed out the potential of H-Phe-Phe-OH motif in nanomedicine (Marchesan et al., 2015b). Multicomponent metallo-nanodrugs as coordination self-assemblies of Fmoc-His-OH and Z-His-Phe-OH in presence of Zn^2+^ ions, formed by the combined efforts of coordination and multiple non-covalent interactions were studied for cooperative coordination of photosensitizer Ce6. These multifunctional nanodrugs with enhanced tumor-specific delivery showed the potential for clinical translation (Li S. et al., 2018).

A controllable self-assembly of H-Phe-Phe-OH in an evaporative dewetting solution has been reported by Chen J. et al. (2015). The all-atom simulations of the small H-Phe-Phe-OH oligomers could accomplish the role of several driving forces and structural motifs that command initial assembly (Jeon et al., 2013). H-Phe-Phe-OH oligomers are associated by hydrophobic interactions between side chains while the H-Phe-Phe-OH zwitterionic peptides having charged termini form ordered, clustered, and compact shapes (electrostatic interactions steer their backbones into a more ordered state as compared to those of uncharged ones). From here on, the hydrophobic interactions of the side chains further lead to higher order oligomers just like in amphiphilic peptides. Therefore, the projected study concludes that the initial precursors of H-Phe-Phe-OH might first assemble into structures that form portions of the packed hydrophobic regions in the nanotube walls (Jeon et al., 2013; Chronopoulou et al., 2014). The aptness of the peptide prompted to explore the mechanical parameters of the H-Phe-Phe-OH self-assembled tubes. Atomic force microscope studies revealed these to be the stiffest (averaged point stiffness of 160 N/m) and with much higher Young's modulus (≈ 9 GPa) as compared to other biological nanostructures, significantly enough to provide mechanical strength to cytoskeleton (Kol et al., 2005). In peptides nanotubes, since the control over length of nanotubes in solution, posed a challenge, a slower co-assembly process along-with the adjustment in the molecular ratio of the H-Phe-Phe-OH assembly unit and its end-capped analog has been suggested (Adler-Abramovich et al., 2016).

The potential of H-Phe-Phe-OH microtubes as intracellular vehicle to release therapeutic compounds has been explored by Silva et al. (2013). The biological marker, rhodamine B, incorporated at the time of self-assembly, had the possibility to intercalate and showed its presence in the inner core to be accepted as intracellular drug delivery for hydrophilic molecules (Silva et al., 2013). These H-Phe-Phe-OH microtubes were further explored for delivering anti-cancer therapeutics, viz., 5-fluorouracil (5-FU) and anti-inflammatory cargo, flufenamic acid (FFA), by Emtiazi et al. (2017) post-conjugation with folic-acid and magnetic nanoparticles. These were found to show the potential as molecular carriers (Emtiazi et al., 2017).

The hydrophobic forces in these well-ordered tubular structures further facilitated to be soluble in a suitable solvent and self-assemble into well-organized films on various substrates. This technique was tried by Zohrabi et al. (2016) wherein FFA loaded inside the H-Phe-Phe-OH nanotubes were coated onto Au surfaces functionalized with 3-mercaptopropionic acid (MPA). The biocompatibility and *in vitro* release studies confirmed the potential of H-Phe-Phe-OH nanotubes as an alternate system for polymer coating in drugs eluting stents. Further, these H-Phe-Phe-OH nanotubes were also shown to possess anti-biofilm activity (Porter et al., 2018). Among the three H-Phe-Phe-OH variants at terminals, such as H-Phe-Phe-OH (l-enantiomer; carboxylic acid terminus), H-*D*-Phe-*D*-Phe-OH (d-enantiomer; carboxylic acid terminus), and H-Phe-Phe-NH_2_, investigated for this purpose, H-Phe-Phe-OH peptide nanotubes were found to be proficient to degrade the biofilm matrix, disrupt cell membranes and hold the potential as efficient drug carrier, though the precise link between these short self-assemblies and biofilm activity needs to be explored.

The peptide-porphyrin macrocycle conjugation has extended its use in biological systems, where the photophysical properties of these molecules and their ability to coordinate metals could be exploited. A review by Biscaglia and Gobbo has presented a survey on biomedical applications of these hybrid compounds, majorly in photodynamic therapy (Biscaglia and Gobbo, 2018). The reversible, biocatalytic and co-assembled nanofibers of Fmoc protected di-peptide (Fmoc-Thr-Leu-NH_2_) and its porphyrin derivative [TCPP, tetrakis(4-carboxyphenyl)porphyrin] advocate the role of Fmoc-moiety in biocatalysts (Wijerathne et al., 2019). The biocompatible photothermal nanodots formed by self-assembly of peptide-porphyrin conjugate; TPP-Phe-Phe-OH (TPP= tetraphenylporphyrin) have been found suitable for tumor ablation and stand good potential for biomedical photoactive applications. The peptide moieties could provide aqueous stability (through hydrophilic interactions) as well as a spatial barrier (through the strong π-π-stacking interactions between porphyrin groups) to inhibit the further growth of light-to-heat converted nanodots with totally inhibited fluorescence emission and singlet oxygen production (Zou et al., 2017).

Self-assembled cationic dipeptides (CDP), H-Phe-Phe-OH, nanocarriers have displayed the ability to encapsulate and transport drug molecules *in vitro* at physiological pH condition by the action of enzymes. A covalent bond formed via Schiff base between oligomeric glutaraldehyde and amino groups of CDP followed by aging yielded CDP nanocarriers (CDPNCs). The π-π interactions of aromatic rings have been proposed to be the driving force for the assembled nanocarriers. Remarkably, the auto-fluorescence due to n-π^*^ transitions of C = N bonds offers visually traceable property in living cells. These highly biocompatible carriers with the ability to encapsulate small guest molecules and enzyme-sensitive nature exhibited a high cytotoxicity against tumor cell proliferation leading to be even used as *in vivo* applications in future (Zhang H. et al., 2015). Though the thermo-induced morphology changes modifying the optical properties in H-Phe-Phe-OH microtubes have been explored by Li et al. (2015), Semin et al. (2015), Nikitin et al. (2016), and Vasilev et al. (2016) the suitability of these H-Phe-Phe-OH based nanocarriers in the intracellular environment of tumor infected tissues was substantiated by Li Q. et al. (2016). Self-assembled hybrid nanospheres, pH- and glutathione (GSH)-responsive, based on H-Phe-Phe-OH and natural alginate dialdehyde as cross linker were used to deliver hydrophobic chemotherapeutic drugs. These nanospheres also contained Au^3+^, reduced to Au *in situ* by ADA, and formed H-Phe-Phe-ADA-Au hybrid nanospheres to facilitate ligand exchange reaction of GSH (Li Q. et al., 2016).

The impact of different degrees of hydrogen bonding and nitrogen substitution on mechanical properties of H-Phe-Phe-OH based peptides, indole-diphenylalanine, N-methyl indole-diphenylalanine, benzimidazolone-diphenylalanine and benzimidazole-diphenylalanine, capped at the N-terminus with heterocycles was executed by Martin et al. (2016). In conjugation with NSAIDs, this change altered the gelator properties. H-Phe-Phe-OH covalently conjugated to NSAIDs was found to form hydrogels and exhibit improved selectivity as compared to the native drug displaying its reputation in drug-delivery (Li et al., 2013). Moreover, the inclusion of ferrocene (Fc) moiety in H-Phe-Phe-OH hydrogel, a redox-active site, could change the morphology from nanosphere to nanofiber with the additional feature to reversibly control the self-assembly process of Fc–Phe-Phe-OH by altering the redox state of the Fc group (Wang et al., 2013b).

The study on impact of different N-terminal capping group to vary the aromaticity on hydrogel formation and properties with glyoxylamide mimics as self-assembly hydrogel by Aldilla et al. (2018) showed that the aromatic caps having the bulky indole side chain of tryptophan and another with electronegative substituent on the phenylalanine ring failed to form a hydrogel; might be because of hindrance in the intramolecular stacking. On the other hand, hydrogels with N-naphthalene sulfonyl cap, exhibited a β-sheet secondary structure with viscoelastic properties and topical delivery of ciprofloxacin and recommended as a drug delivery vehicle. Since H-Phe-Phe-OH forms nanofibers with remarkable optical and electrical properties, optical microscopy methods, fluorescence microscopy and super resolution single molecule localization microscopy have been suggested for the study of H-Phe-Phe-OH –based nanostructures (Pujals et al., 2017).

Kuang et al. made an attempt to establish the difference in properties between supramolecular nanofiber assemblies of small molecules and the individual molecules on interaction with the cells. The study of nanofibers formed by the self-assembly of two phenylalanine residues and a naphthyl group, with β-sheet-like-structure revealed the threshold concentration for the formation of nanofibers and suggested hydrophobicity to be responsible for higher cytotoxicity as compared to unassembled monomers (Kuang and Xu, 2013). A photo-responsive hydrogelator, wherein, H-Phe-Phe-OH was protected by 6-nitroveratryloxycarbonyl moiety; Nvoc-Phe-Phe-OH, has been designed and synthesized by Roth-Konforti et al. (2018). The photo-labile trigger, 4,5-dimethoxy-2-nitrobenzyl alcohol, served as a π-π stacking element in place of the aromatic Fmoc group. This stiff 3D-hydrogel displayed responsiveness to UV light irradiation and it completely disassembled subsequently on irradiation at room temperature to release the entrapped drug at the site of administration. The patterning of the photo-responsive peptide based hydrogel along with gradual release of insulin-FITC conferred linear correlation to the stimulus duration. Fmoc-Phe-Phe-OH gel remained unaffected by UV irradiation and showed insignificant FITC-insulin release post-UV application (Roth-Konforti et al., 2018).

The self-supporting gel, Fmoc-Phe-Phe-OH, an ultra-short peptide, is one of the most studied hydrogel. Fmoc-Phe-Phe-OH peptide has been reported by Aviv et al. to deliberate the mechanical rigidity and stability to hyaluronic acid (HA), a major component of the extracellular matrix, without the use of molecules to initiate chemical cross-linking. The Fmoc-Phe-Phe-OH/HA hydrogel, composed of two components, could allow fine-tuning of the hydrogel parameters, adjusted for injection and malleable and facilitating its use in drug-delivery and tissue engineering applications (Aviv et al., 2018). A review by Diaferia et al. describes the immense potential of the hydrogel specially in the field of drug delivery, tissue-engineering and catalytic behavior (Diaferia et al., 2019). Fmoc-Phe-Phe-OH based hydrogel nanoparticles have been explored as nano-carriers to encapsulate and deliver drugs/bioactive molecules. A scalable process for the assembly of Fmoc-Phe-Phe-OH peptide into hydrogel nanoparticles as ensuing drug delivery carriers has been outlined by Ischakov et al. (2013) The results of encapsulated Dox and 5-FU with different structural characteristics endorsed the assumption that the encapsulation ability of nanoparticles could be dependent upon the physicochemical nature of the molecules, thus affecting the release kinetics. Erdogan et al. have used freeze-quenching technique to prepare plasmonic nanoparticle-embedded organogels from Fmoc-Phe-Phe-OH and gold nanorods for efficient drug delivery. The controlled and enhanced release of the Dox was manipulated using laser illumination (Erdogan et al., 2016). The study by Truong et al. (2015) on Fmoc-Phe-Phe-OH self-assembled hydrogels with 5-FU and paclitaxel cautioned that it is the leaching time, not the exposure time which affects the overall cell viability and thus the cytotoxic effects could be observed only after the gel is completely dissolved. Since more stable gels are prone to leach their monomers slower into the neighboring biological environment, the overall concentration of these monomers and any potential adverse effects get reduced. Therefore, the factors controlling the stability of Fmoc-Phe-Phe-OH self-assembled hydrogels are more critical to continue this field (Truong et al., 2015). Argudo et al. have discussed the rules for chemical design through selection of the amino-acid sequence of Fmoc-dipeptides with the self assembling capability for 2D self-assembly at the air/water interface to form supramolecular structures (Argudo et al., 2018). The values of log P and -log S parameters have been proposed based on experimental results which can predict the possibility for a particular Fmoc-dipeptide to self-assemble at fluid interface. A cellular biosensing system, based on 3D culture model using Fmoc-Phe-Phe-OH dipeptide hydrogel employed as both, a 3D cell culture scaffold to provide a confinement in environment and an immobilized enzyme matrix through simple one-pot self-assembly is suggested for the detection of superoxide anion (O•-) by Lian et al. (2017).

A non-centro-symmetric β-sheet structure in Fmoc-Phe-Phe-OH nanofibrils, branded as biomimetic materials with mechanical properties equivalent to the biological gels, also displayed piezoelectric behavior which could enable these assemblies as scaffolds for tissue-engineering (Ryan et al., 2015). Fmoc-Phe-Phe-OH, hydrogel matrix, has been used by Lian et al. to construct a smart biointerface, followed by enzyme-based electrochemical biosensing and cell monitoring. The encapsulation of horseradish peroxidase (HRP) was achieved during self-assembly, which was subsequently used to detect the release of H_2_O_2_ from living cells (Lian et al., 2016).

**Figure 5:**
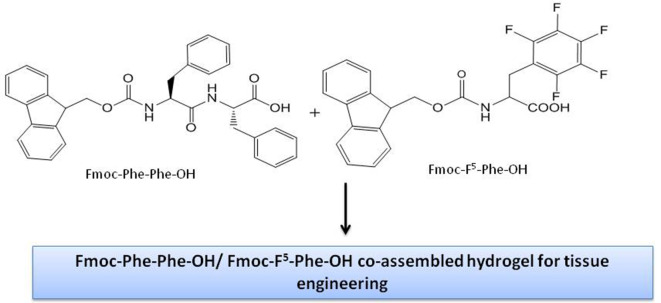
Multicomponent co-assembly to yield ultra-rigid hydrogel.

The synergistic effect of the co-assembly of Fmoc-F^5^-Phe and Fmoc-Phe-Phe-OH has been reported to result in a ultra-rigid hydrogel with controllable mechanical properties optimum for tissue engineering (Figure 5) (Halperin-Sternfeld et al., 2017). The morphological transition from hybrid nanospheres to visible macroscopic films in the co-assembly of the cationic dipeptide (CDP), H-Phe-Phe-NH_2_ • HCl and a Keggin-type polyoxometalate (POM), phosphotungstic acid (PTA), upon photothermal treatment was observed which could be suitable for tissue engineering (Xing et al., 2015).

The *in vivo* studies of self-assembled, injectable, fibrous hydrogels obtained by the combination of dipeptide Fmoc-Phe-Phe-OH and poly-L-lysine have been reported to deliver the photosensitive drug Ce6 in a controlled manner at the tumor site and are suggested to work well under the strategy “once injection, multiple-treatments” by Abbas et al. (2018). Taking in and away plan of sheer forces has been observed to work well for the transmission from gel to sol state supporting the self-healing behavior.

Lately, the study by micro-second molecular dynamics simulations on the co-assembly of H-Phe-Phe-OH with different types of non-H-Phe-Phe-OH dipeptides manifested regular-shaped vesicles, single-/multi-cavity assemblies, and planar sheets, which otherwise, are hardly observed in self-assemblies of non-H-Phe-Phe-OH dipeptides. The balancing act between electrostatic repulsion, hydrophobic and aromatic stacking interactions attributes toward the formation of varied structures (Tang et al., 2020). Another novel, multi- component, organic- inorganic peptide-based hydrogel, with two building blocks, viz., Fmoc-Phe-Phe-OH and Fmoc-Arg-OH, and an inorganic material, hydroxyapatite (HAP) as 3D scaffolds was designed for bone tissue regeneration. Fmoc-Phe-Phe-OH served as rigid hydrogel that could mimic the ECM, arginine aided in tissue development and might also be helpful to fine tune the bioactivity of HAP, while HAP itself promoted the mechanical properties of the composite material. An optimum composition of the three resulted in a rigid and biocompatible hybrid suitable for cell adhesion and a bone tissue engineering scaffold (Ghosh et al., 2017).

A multicomponent dipeptide hydrogel from Fmoc-3F-Phe-Arg-NH_2_ and Fmoc-3F-Phe-Asp-OH, without covalent connection between Arg and Asp, co-assembled by aromatic, hydrophobic, and coulombic interactions displayed the feasibility in the design of innovative materials for tissue engineering (Liyanage et al., 2015).

It had been established that removal of the N-terminus charge via addition of a Boc group could not stop the formation of H-Phe-Phe-OH like structures (Levin et al., 2014). Boc-Phe-Phe-OH in different solvent conditions was observed to display tuneable structures of two different morphologies: tubular or spherical under different solvent conditions and the presence/absence of superparamagnetic iron oxide (SPIONs) core and studied for dual use (drug delivery and heat generation) (Majid et al., 2017). Also, molecular dynamics simulations had suggested that the N-terminus stabilizes the initial stage aggregates during the self-assembly formation while in the final tube structure, it does not rely on the charged termini regions. So when a mixture of H-Phe-Phe-OH and Boc-Phe-Phe-OH monomers, where the uncharged Boc group was added to the N-terminus of the dipeptide, leaving the aromatic residues free to interact, the fibers with varying morphologies and mechanical properties were grown depending on the ratio of the two monomeric dipeptides. Atomic force microscopy (AFM), fluorescence and FTIR spectroscopy showed a reduced Young's modulus and rigidity as well as reduction in hydrogen bonding along with an alteration in the electronic state of the aromatic residues in co-assembled fibers. So it was hypothesized that this reduction in the hydrogen bonding and a modification in π-π stacking between monomers altered the packing co-assembly. So, the study gives clue to develop various materials with different surface groups resulting in different interactions impacting the mechanical properties of scaffolds to be used in tissue-engineering (Creasey et al., 2016).

The co-assembly of H-Phe-Phe-OH and Boc-Phe-Phe-OH formed the fibers in aqueous conditions with reduced stiffness and curvier morphology. Creasey et al. proposed the disruption of the hydrogen bonding among neighboring N-termini and a variation of π-π stacking between monomers altered the packing of co-assembly and thus provided an option of post-assembly chemical functionality and supramolecular sites for drug delivery vehicles (Creasey et al., 2016). Just to briefly mention, a new prototype, an orthogonal strategy, for the cargo release from micro-compartments by modulating the intrinsic self-assembly state and opening the prospects to use the triggered self-assembly to finally control the transport of various molecular species across interfaces has been demonstrated with Boc-Phe-Phe-OH, which could trigger jet-like release within seconds (Levin et al., 2018). Among the short hybrid dipeptides composed of β(O)-δ5 -amino acids synthesized by Reja et al., the injectable hydrogel, Boc-β(O)-δ5-Phe-β(O)-δ5-Phe showed its utility in 2D-cell culture and could encapsulate proflavine solution in the gel matrix and release slowly (Reja et al., 2019).

Rhein (4,5-dihydroxyanthraquinone-2-carboxylic acid), a traditional Chinese anti-inflammatory compound, exhibiting planar aromatic structure, is branded to enhance intramolecular π-π stacking in dipeptide-based materials. The rhein-Phe-Phe-OH peptide gets muddled into uniform spherical nanoassemblies spontaneously, suggesting its competence as carrier for drug delivery. The virtual screening of rhein-Phe-Phe-OH from Traditional Chinese Medicine (TCM) database (based on MD simulations), showed its significant binding energy toward camptothecin (CPT), known for strong antitumor efficacy but limited in use due to poor solubility and stability (perhaps the first compound to be designed cooperatively by both experimental studies and computation simulations). *In vitro* and *in vivo* experimental evaluations validated the computation simulations (Sun et al., 2019).

Amoc-Leu-Phe-OH; an Amoc-capped dipeptide, wherein, Amoc moiety is supposed to provide optimal hydrophobicity, which in turn, could enhance self-assembling propensity demonstrated anti-inflammatory action using the rat air pouch model and antibacterial efficiency against Gram-positive and Gram-negative bacteria. The non-cytotoxic hydrogel has been suggested to be used in targeted drug/cell delivery and for wound healing applications by Gavel et al. (2018b).

Non-proteinogenic α,β-didehydro-α-amino acids, with peculiar conformational properties- planar conformation due to a double bond, between Cα and Cβ carbon atoms and fixed values of t ϕ and ψ torsion angles, have shown their ability to form hydrogels (Vilaça et al., 2015b) and applicable in drug delivery. A procedure for cheaper, less laborious and less time taken than other methods of dehydropeptide synthesis (α,β-didehydro-α-amino acid) through oxidation of the C-terminal residue in N-Boc-protected and N-Fmoc-protected peptides, both in solution and on the solid support for peptide synthesis has been reported by Wołczanski and Lisowski (Wołczanski and Lisowski, 2018). The ability to form secondary and super secondary structures and highly stable nanostructures has made α,β-didehydrophenylalanine a center of attraction for the researchers (Gupta and Chauhan, 2011). Self-assembled nanoparticles of dipeptide, methionine-dehydrophenylalanine (Met-ΔPhe), having an unsaturated analog of naturally occurring phenylalanine to entrap curcumin, an anticancer agent, have been synthesized and used by Alam et al. (2012). In this study, the role of ΔPhe in self-assembly of H-Met-ΔPhe-OH was compared with the self-assembly of H-Met-Phe-OH having natural L-phenylalanine. The comparison signified that the presence of ΔPhe in H-Met-ΔPhe-OH promoted the formation and stability of self-assembled dipeptide NPs remarkably, and enhanced entrapment efficiency of curcumin. Besides, the combination of dipeptide assembly with curcumin offered several additional advantages such as high biocompatibility of the former, inhibition of the tumor growth and increased longevity of melanoma tumor-bearing animals of the later. Self-assembly of the dipeptide not only demonstrated the entrapment of curcumin, which could not be used up to its potential due to its hydrophobic nature, leading to poor aqueous solubility, but also improved its bioavailability. Therefore, such dipeptide-NPs could also be used to improve the delivery of other effective hydrophobic drug molecules. Further work on the self-assembled nanotubes using two dipeptides with non-natural amino acids, one containing a flexible H-βPhe-Phe-OH amino acid (βPhe–Phe) and the other containing both H-βPhe-Phe-OH as well as a backbone constrained α,β-didehydrophenylalanine amino acid, H-βPhe–ΔPhe-OH, (βPhe–ΔPhe), was reported to have different properties than the native H-Phe–Phe-OH nanotubes. The study also addressed the effect of backbone length, introduction of conformational flexibility (caused by β-Phe) and constraint (produced by ΔPhe) on the self-assembly process and morphology. The dipeptide crystal, H-βPhe–Phe-OH, showed two conformations, viz., a gauche conformer forming the channel and a trans conformer trapped inside the channel formed by the gauche conformer, probably the first example presenting such self-entrapment. The nanotubes could efficiently encapsulate small hydrophobic drug molecules. The efficient delivery of anti-cancerous drug, mitoxantrone, entrapped in nanotubes as compared to free mitoxantrone can lead to development of more biocompatible and proteolytically stable drug delivery vehicles (Parween et al., 2014). Similarly, H-Phe–ΔPhe-OH based nanotubular system as sustained release nanocarriers for intravitreal delivery of the angiogenic drug was investigated. The sustained delivery potential, as pre-loaded and post-loaded options during nanotube formation assembly, was compared to the plain drug. It was speculated that in post-loading approach, there was a possibility of hydrophobic and aromatic π- π stacking interactions between the drug and the dipeptide. Additionally, certain amount of dipeptide might have released from the nanotube surface which could further entrap the drug within the nanotubes. The hydrophobic nature of the drug speculates its participation in the development of ordered nanotubes. Phenylalanine-α,β-didehydrophenylalanine (Phe-ΔPhe) dipeptide nanotubes were also explored by Khatri et al. to deliver cancer-testis antigens (CTAs). H-Phe-ΔPhe-CTA nanocomplex showed an inhibition in tumor growth in animal studies, opening the door for this dipeptide as *in vivo* delivery vehicle for CTAs (Khatri et al., 2019).

An arginine containing dipeptide, arginine-α,β-didehydrophenylalanine (Arg-ΔPhe), showed the capability to condense the plasmid DNA into positively charged nanoparticles of spherical morphology and deliver it inside the cells and distribute in cytosol and endosomes. The methodology offers simple synthesis route and characterization, viz., stability, biocompatibility and significant transfection efficiency, and made H-Arg-ΔPhe-OH a suitable contender for *in vivo* gene delivery applications (Khatri et al., 2017). The introduction of non-protein amino acid, namely N-methylated amino acid, *D*-amino acid and α,β,-didehydroamino acid, in peptides provides enhanced stability with regard to enzymatic degradation. Self-assembled nanoparticles of H-Arg-ΔPhe-OH were synthesized, characterized and derivatized with folic acid which showed high affinity for its cell surface receptors. The results exhibited significant cellular uptake and tumor regression toward different cancer cells compared to free drug, Dox /drug-loaded underivatized NPs both *in vitro* and *in vivo*. Iron oxide nanoparticles (IONP) conjugated H-Arg-ΔPhe-OH also showed heat-induced cytotoxicity on lung cancer cells which resulted in the death of these cells (Baskar et al., 2017). Further, it was demonstrated that the presence of arginine in the cationic dipeptide nanoparticles endorsed pH responsive behavior. FTIR and XRD analysis results for compatibility assessment ensured the retention of Dox activity and efficiency within the developed H-Arg-ΔPhe-Dox NPs. pH dependent release studies showed signs of maximum release at acidic pH and so, an excellent choice for site-specific drug delivery in tumors and tissues harboring acidic microenvironment like that of stomach (Singh et al., 2018). The H-Arg-ΔPhe-OH NPs, when conjugated with lactobionic acid (LA), a ligand for the asialoglycoprotein receptors, overexpressed in hepatocellular carcinoma (HCC) cell lines, achieved targeted delivery. H-Arg-ΔPhe-LA/miR NPs demonstrated the selective delivery of miR-199a-3p and so, the formulation could be used in HCC therapy (Varshney et al., 2018).

Spontaneous self-assembly of a dipeptide, leucine-α,β-didehydrophenylalanine (Leu-ΔPhe), into a stable hydrogel, was observed to enhance the antitumor activity of the drug, mitoxantrone. The amphipathic nature of the hydrogel, due to unprotected N- and C-terminals in H-Leu-ΔPhe-OH, could lend polar character. The side chains, bearing a strong hydrophobic character, offer the recipe for the entrapment of both hydrophilic and hydrophobic drugs in the gel matrix and release in a controlled manner (Thota et al., 2016).

Discrete nanoparticles synthesized by combination of different plasmid DNAs with cationic dipeptides. H-Lys-ΔPhe-OH and H-Arg-ΔPhe-OH, showed the potential of these dipeptides to protect DNA from enzymatic degradation and carried them to cellular cytoplasm and nucleus. The results showed that the DNA was packaged inside peptide NPs with no visible cytotoxic effect and exhibited enhanced cellular uptake. Therefore, these could be influential in non-viral mediated gene delivery applications (Panda et al., 2013).

Vilaça et al. synthesized naproxen N-conjugated dehydrodipeptide hydrogelators, having at least one aromatic amino acid (Vilaça et al., 2015b). Also, the evaluation of tryptophan N-capped naproxen and C-terminal dehydrophenylalanine (ΔPhe), dehydroaminobutyric acid (ΔAbu), and dehydroalanine (ΔAla), as protease resistant hydrogelators, demonstrated that Npx-Trp-*Z*-ΔPhe-OH (with C-terminal ΔPhe) was found to be the most suitable nanocarrier among the lot for the delivery of encapsulated low molecular weight drugs (Vilaça et al., 2015a). These results supported the development of a peptide construct, Npx-Ala-*Z*-ΔPhe-Gly-Arg-Gly-Asp-Gly-OH, which was also demonstrated to have properties of an efficient drug carrier (Vilaça et al., 2017). Further studies with tyrosine and aspartic acid residues, tyrosyldehydrophenylalanine [Npx-Tyr-*Z*-ΔPhe-OH] and aspartyldehydrophenyl-alanine [Npx-Asp-*Z*-ΔPhe-OH], showed that the incorporation of superparamagnetic iron oxide nanoparticles (SPIONs) could keep the magnetic behavior alive in spite of the strong diamagnetic contribution from the involved organic matrix. *In vitro* heat generation on magnetic excitation with AMF could convert the gel to a solution, acting as a remote trigger to release SPIONs and encapsulated drugs displaying an image-guided drug delivery through self-reporting mechanism (Carvalho et al., 2019). Recently, a newer dehydropeptide hydrogelator; Npx-Met-*Z*-ΔPhe-OH (containing naproxen and a thioether), was combined with two different architectures, core/shell manganese ferrite/gold nanoparticle and gold-decorated manganese ferrite nanoparticles (acclaiming the affinity of sulfur toward gold) and tested for the delivery of antitumor drug, curcumin by the group (Veloso et al., 2020). ΔPhe is known to endow proteolytic stability and add conformational restraints in the peptide backbone. The naproxen group is added to boost selectivity toward cyclooxygenase. The drug delivery was higher in both irradiated gels as compared to the non-irradiated gels, suggestive for multimodal cancer therapy by combining both the photothermia and controlled drug delivery.

Different metal ions as well as anions modulating the self-assembly of the peptide amphiphiles have been investigated by Sharma et al. advocating an ion responsive behavior to the hydrogels. The group (Sharma et al., 2019) has reported various metal salts to influence self-assembly of histidine based dipeptide amphiphile accompanying a gel to sol transition. In another study, Fan et al. have shown the capability of a dipeptide, H-Trp-Phe-OH, nanoparticles conjugated with an aptamer to bind Dox, due to the π-π stacking interaction and Zn(II) chelation. The formulation exhibited the possibility to act as optical probe to monitor the drug uptake and release (Fan et al., 2016). As recommended by Gazit, the study on fluorophores derived from green fluorescent protein and dipeptide conjugates sets the stage for new frontiers in peptide optics and to load various aromatic drug molecules into these nanoparticles via aromatic stacking interactions (Gazit, 2016).

The nanotubes of H-Lys-Lys-OH functionalized with camptothecin (CPT) chromophore at the ε-amino position, Ac-Lys-Lys(CPT)-NH_2_ and NH_2_-Lys-Lys-(CPT)-NH_2_, were observed to protect the drug from lactone hydrolysis by sequestering the CPT segment within the hydrophobic nanotube walls, thereby increasing its stability. Herein, CPT conjugated dipeptide played the dual function, both as drug and precursor to the nanostructured carrier (Kim et al., 2015). Similar to this scheme, the research group Sun et al. worked for the delivery of 5-FU and synthesized di-lysine conjugated with 5-fluorouracil (5-FU), wherein hydrophobic π-π association of both uracil and Fmoc could drive β-sheet self-assembly (Sun Y. et al., 2016).

Fluorescein isothiocyanate (FITC) is known as sensitive fluorescent probe which can be detected easily in living cells. The attachment of fluorescein to N-terminal of H-Leu-Leu-OH dipeptide carried out by Kirkham et al. has found to be more cytocompatible as compared to the molecule itself. The strong π-π stacking interactions between the aromatic units in conjugate molecule, Fl-Leu-Leu-OH, formed could be responsible in the induction of self-assembly process. Fl-Leu-Leu-OH adopted a β-sheet structure as shown by FTIR spectroscopy, cryogenic transmission electron microscopy (cryo-TEM) and small-angle X-ray scattering (SAXS) studies. The conjugation improved the capacity of Fl-Leu-Leu-OH to be uptaken by cells without compromising cell viability as compared to FITC alone (Kirkham et al., 2016).

Use of a dipeptide, H-Asp-Phe-OH, to assist the uptake of a therapeutic polypeptide drug, calcitonin, broadly used to treat bone diseases, has been demonstrated by Cao et al. (2017) as co-assembly of the peptide-drug nanoparticles. The dipeptide (Asp-Phe) simply interacted with the Salmon calcitonin (sCT) through hydrophobic and electrostatic interactions leading to the supramolecular nanoparticles of sCT-Asp-Phe-OH. This self-assembly established long-lasting therapeutic effect and could control *in vivo* release of sCT, thereby, shun the need for multiple injections in patients (Cao et al., 2017).

The formation of NDI-Tyr-Phe-NH_2_ by thermodynamically driven enzymatic condensation of naphthalenediimide (NDI)-functionalized tyrosine (NDI-Tyr) and phenylalanine-amide (Phe-NH_2_) has been reported by Nalluri et al. (2014). The presence of dihydroxy/alkoxy naphthalene donors could produce proficient charge-transfer complexes. The fully reversible self-assemblies driven on minimized free-energy and fewer defects holds potential to develop self-healing materials.

Microvesicles, formed from a water-soluble, synthetic, amphiphilic dipeptide, containing a glutamic acid residue at the C-terminus and hydrophobic residue (Val/Leu/Nva) at N-terminal have been established to deliver and release the encapsulated anticancer drug and a fluorescent dye in response to a stimulus of the presence of calcium ions. The dye/drug-loaded vesicles have been found to be non-toxic using 2-(4,5-dimethyl-1,3-thiazol-2-yl)-3,5-diphenyl-2,3-dihydro-1H-tetrazol-4-ium bromide (MTT) based cytotoxicity assay. These biocompatible microvesicles were reported to have the capability to carry cyclic adenosine monophosphate (cAMP) at the target site suggesting the potential of these delivery vectors to carry drugs and other bioactive molecules (Naskar et al., 2011).

A dipeptide hydrogel, containing double Fmoc-group, self-assembled at very low concentration, exhibiting two-step self-assembly process, cytotoxic, suitable for 2D/3D cell scaffolding has been reported recently. The self-assembly of Fmoc-Lys(Fmoc)-Asp-H is reported to have gained advantage from (i) additional H-bonding from the carbonyl group, (ii) aromatic and hydrophobic interactions from the fluorenyl ring, and (iii) steric optimization from the methoxycarbonyl moiety. The balance between the hydrophobic and hydrophilic functionalities is essential for the self-assembly. So the hydrophobicity due to the additional Fmoc group is suggested to be balanced by the two hydrophilic carboxylic acid moieties present at the C-terminus as well as the Asp side chain (Chakraborty et al., 2020).

As linkers play an important role in speedy and effective delivery of the cytotoxic drugs, a lysosomal protease-cleavable dipeptide H-Val-Cit-OH, has commonly been used in the designing and synthesis of antibody-drug conjugates (ADCs) for delivering cytotoxic drugs to antigen bearing cells. Besides, H-Val-Ala-OH, another important dipeptide, has also been employed for synthesizing next-generation ADCs useful for drug loading and releasing purposes. The comparison studies of H-Val-Cit-OH and H-Val-Ala-OH based ADCs conjugated to the monomethyl auristatin E(MMAE) payload using xenograft model test showed their ability as patent payload for the anti-tumor activity (Wang et al., 2017b; Wang W. et al., 2017). It has also been reported that adding a hydrophilic group to the N-terminus of the valine residue in the H-Val-Cit-OH linker, viz., glutamic acid, prevents the premature cleavage of the linker in mouse plasma which otherwise is susceptible to extracellular carboxylesterase. This enhancement in the polarity of H-Val-Cit-OH linker offers higher *in vivo* stability to the antibody–drug conjugate in mouse plasma and allows the designing of ADC linkers (Anami et al., 2018).

An amphiphilic dendron, N-octadecanoyl-1,5-bis(L-glutamic acid)-L-glutamic diamide with potential to entrap Vitamin B_1_ (VB_1_) was found to shrink and turn into supergel triggered by monovalent to tetravalent metal ions. The mechanism of continuous, pH-responsive and thermally reversible shrinkage process has been proposed to involve protonation of carboxylic acid groups in presence of metal ions transforming morphology of the hydrogel from nanotubes to nanobelts followed by crosslinking between protonated carboxylic acid groups and metal ions again transforming morphology to nanofibres. During this transition process, the encapsulated water was released but immobilized due to cross-linked 3D structures and VB_1_ was released in water phase. The release rate was found to be tuneable upon the shrinking rate of OGAc/metal hydrogel arrangement (Qin et al., 2013). Since the best shrinkage capability was exhibited by Mg^2+^ ions, this research group used the proposed phase regulation property with change in pH in a further study. It was also observed that the addition of positively charged species accelerated the shrinkage and negatively charged additives suppressed the shrinkage of the supramolecular hydrogel. These pH change and ionic separation properties were exploited for the step-wise release of two-component drugs, pralidoxime iodide and phenol red, first the anionic drug, phenol red, was released through gel shrinkage and then on increasing pH, released the second cationic drug, pralidoxime iodide, through gel collapse, which suggests that the system still demands more sophisticated work to be carried out on the concept (Qin et al., 2014). Another pH- responsive hydrogel from the class of N-acetylglucosamine derivatives, especially, the heptylurea derivative with a *p*-methoxybenzylideneacetal protective group, showed release of trapped naproxen on addition of acid (Goyal et al., 2014).

Cyclic peptides have advantage to linear peptides in terms of structure rigidness and stability against proteolytic enzymes. This structure rigidity can reduce the freedom of possible structural conformations and so enhance the binding affinity of the ligands toward receptors. The cyclo-*D*-Trp-Tyr peptide nanotubes have been synthesized and assessed as carriers of caspase 3 silence shRNA. This could penetrate the intact cornea and deliver the CAP3 pRFP-C-RS DNA reducing the apoptosis in the wounded cornea triggered by corneal epithelial debridement (Lee et al., 2015). The injectable, *in situ* ambidextrous supergelator, cyclo-Phe-Glu(O-*tert*-But) with cytocompatibility has been reported to hold potential in drug delivery and regenerative medicine (Manchineella et al., 2017). A cationic, cyclic dipeptide, photoresponsive hydrogelator, with a diketopiperazine (DKP) derivative containing an unnatural amino acid bearing an azobenzene photoswitch (PAP–DKP); PAP-DKP-Lys favorably encapsulated oligonucleotides and released upon irradiation, thereby showing the potential for tuning the amino acid residue for the particular cargoes (Pianowski et al., 2016).

### Tripeptides as Biologics Carriers

Amino acid chirality, considered a key tool to drive peptide self-assembly, exploited at the molecular and supramolecular levels for *D*- and *L*-amino acids positions in case of gelling tripeptides has been reported by Marchesan et al. (2015a), to establish a higher or lower supramolecular order. So, a suitable design of the chirality in sequence of self-assemblies has been suggested to assign and fine-tune the properties of reported tripeptide gel biomaterials. The amphiphilic tripeptides have been reported to generate diverse nanostructures with variable aspect-ratio and geometry as the amino acid is altered by Matson and Stupp. Hydrogen bonding between amide proton donors and adjacent carbonyl proton acceptors leading to β-sheet-type association in self-assembling oligopeptides has been found to favor the formation of high aspect-ratio nanostructures (Matson and Stupp, 2012). Sahoo et al. carried the gelation of tripeptides, by connecting a hydrophobic phenylalanine residue at the N-terminus, a variable amino acid to Ac-Phe-X-Asp-NH_2_ to alter the tendency for intermolecular hydrogen bonding [X= G/A/V/L/I residue], and a charged C-terminal aspartic acid residue. They found that even without a strong peptide-specific secondary structure, the tripeptides with last three of the mentioned amino acids formed hydrogels (Sahoo et al., 2017). Furthermore, the work on selected Ac-Phe-X-Asp-NH_2_ sequence with high aspect ratio; Ac-Phe-Ile-Asp-NH_2_, suggested the requirement of the non-aromatic and uncharged residue in this tripeptide, which in this case was isoleucine–to be at least hydrophobic as well to facilitated high aspect-ratio nanostructure and aid hydrogel formation (Sahoo et al., 2018a). The study by Yang et al., on structural transition in Fmoc-Phe-Phe-Cys-OH from parallel-aligned worm-like micelles to entangled, coiled micelles persuaded by the cross-linking of the disulfide bonding, elucidated the relationship between micelle chirality and gel properties and suggested a strategy to fabricate materials with controlled chirality by means of synchronized non-covalent and covalent self-assembly (Yang et al., 2019). A series of Fmoc- protected tripeptide containing β-amino acid from H-βAla-His-OH [L-carnosine (Car)] joined by covalent link to different Fmoc-*L*-amino acids with a wide range of side-chains having different hydrophobicities were observed by Das et al. The tripeptides were found to be proteolytically stable under physiological condition and produce helical structures and the gelation capability to depend on the hydrophobicity of the side chain moiety where the additional π-π stacking interaction by the phenyl group supported the process. This study also demonstrated that the Fmoc-Phe-βAla-His-OH containing Fmoc-tripeptide exhibited the highest gelation ability among the examined amino acid residues, may be the phenyl group provided π-π stacking interaction. The tyrosine residue, expected for similar associative interaction, showed less gelation ability due to intermolecular H-bonding through the phenolic –OH group (Das Mahapatra et al., 2017).

Two tripeptides, viz., H-Gly-His-Lys-OH and H-Phe-Phe-Asp-OH, co-assembled through non-covalent assimilation, easily formed complex selectively with copper ions and converted into a hydrogel formed of nanofibers from clear solution of nanotapes in response to complexation (Abul-Haija et al., 2017).

A tripeptide, H-Lys-Phe-Gly-OH, accomplished the morphology change in secondary structure with switching of nanostructures from nanospheres (vesicles) to nanotubes reversibly depending on the concentration. A transition in the secondary structure could change the morphology of nanostructures. At the low concentration, the tripeptide formed spherical structures with diameters of 50±10 nm, while, at the higher concentration, the tubular aggregates with diameters of 190±10 nm were observed by Moitra et al. (2014, 2017). Even a temperature-induced change from tubular to vesicular has been observed. The nanovesicles, formed at physiological pH and disrupted at pH 6, a fit condition for the drug-delivery, were loaded with a chemotherapeutic anticancer drug, Dox, which resulted in the intracellular release of the drug.

The self-assembled nanoparticles of Fmoc-Leu-Leu-Leu-OMe have been reported by Li et al. as carriers of hydrophobic porphyrin derivative, meso-tetra(p-hydroxyphenyl) porphine (m-THPP) (Li J. et al., 2018). The doping of peptide nanoparticles facilitated the solubility of hydrophobic porphyrin in aqueous media. Additionally, the doping preserved the intrinsic fluorescent property and reduced the risk of side effects due to overdose. The doping could enhance the two-photon fluorescence absorption ability of m-THPP. *In vitro* reactive oxygen species detection and the cytotoxicity test suggested high anticancer efficacy of the peptide nanoparticles by the two-photon irradiation (Li J. et al., 2018; Li et al., 2019a).

**Figure 6:**
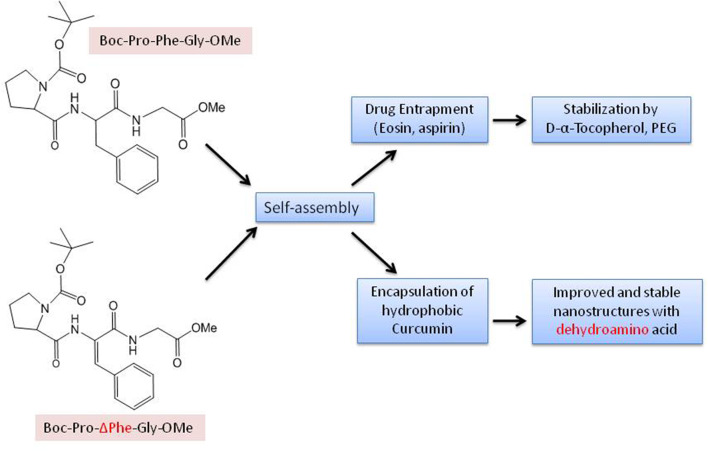
Self-assembly of tripeptides having different aromatic moities.

Fmoc-halogenated phenylalanine hydrogels have also been prepared and reported by Wang et al. (2013a). Out of various fluorinated analogs, Fmoc-4F-Phe-OH was found to be most suitable since the fluorenyl groups stacked more extensively. However, none of these hydrogels were found suitable to serve as a bioactive scaffold for NIH 3T3 cell culture in 2D environments. Thereafter, H-Arg-Gly-Asp-OH peptide was included in Fmoc-4F-Phe-OH to aid the cells' survival. This compound showed 3T3 cell adhesion and competent cell division. The results were suggestive of using similar bioactive scaffolds for drug delivery, tissue engineering and cell culture etc (Wang et al., 2013a). A self-assembled protected tripeptide, containing hydrophobic core, Boc-Pro-Phe-Gly-OMe, showed the ability to encapsulate hydrophobic drugs—eosin, aspirin and curcumin. The stabilization of the encapsulated nanostructures with Vitamin E-TPGS illustrated the controlled release of drugs endorsing their role as drug carriers (Yadav et al., 2015). Later, replacement of phenylalanine with dehydrophenylalanine, a constrained dehydroamino acid, self-assembled as hydrophobic matrix dehydrotripeptide, Boc-Pro-ΔPhe-Gly-OMe. The enhanced encapsulation competence of hydrophobic molecules showed soundness of constrained dehydroamino acids in drug-delivery applications (Figure 6) (Deka et al., 2017).

H-Phe-Phe-OH could promote self-assembly and gelation through π-π and hydrophobic interactions to construct gel materials. Also, an amino acid, 11-aminoundecanoic acid (AUDA), non-proteinaceous with long aliphatic chain, could add thixotropic property to the hydrogel. A combination of AUDA and H-Phe-Phe-OH residues as Boc-AUDA-Phe-Phe-COOH could promote both hydrogelation and thixotropic nature. The resultant hydrogel exhibited encapsulation and sustained release of an antibiotic (vancomycin) and vitamin B_12_ at physiological pH and temperature and endorsed itself for drug-delivery applications (Baral et al., 2014).

High-resolution scanning electron microscopic analysis revealed the architectural differences in the synthesized tripeptides, Boc-Phe-Gly-Glu-OH (L_1_) and Boc-Phe-Val-Glu-OH (L_2_), differing in hydrophobic groups and their corresponding Cu(II) conjugates (Das et al., 2018). The tripeptide with more hydrophobic glycine generated flower-like decorated branches and the less hydrophobic formed well-ordered spherical assemblies with nano-meric dimensions. The corresponding metallic conjugates self-assembled as nano-belt like structures in the former and nano-flake like in the later, which could be due to the metal–peptide interactions that controlled the self-assembly process, entailing that the metal–peptide interactions settle on the morphology of the assemblies. The observation on these non-cytotoxic metallo-peptides; Boc-Phe-Gly-Glu-Cu (L_1_M) and Boc-Phe-Val-Glu-Cu (L_2_M), could establish the role of metal ions- peptides coordination in removal of strong non-covalent interaction and the formation of supramolecular architectures with various morphologies (Das et al., 2018).

Fmoc-tripeptide hydrogels from Fmoc-Phe–Phe–Phe-OH with different chirality were synthesized by Chronopoulou et al. by a lipase-supported reaction in an aqueous phase using genipin as a crosslinker. The proposed crosslinker could modulate the physicochemical features of such hydrogels. Hydrogels displayed release of almost same amount of the entrapped drug, Dexamethasone (DXM), though the release kinetics varied. The *D*-amino acid based hydrogels were observed to be “dense” materials, so could detain the entrapped drugs for longer period than the corresponding *L*-counterparts and could also resist protease degradation. The results could establish the importance of chirality to induce the specific secondary structural features and also modulate physicochemical properties of the hydrogel. These biomaterials hold bright future in drug delivery and scaffolding uses in tissue engineering (Chronopoulou et al., 2014, 2015, 2017).

The prevalent challenges among cell-scaffolding materials are to upkeep with the vital physiognomies of the otherwise complex natural extracellular matrix (ECM) and incorporate the essential characteristics with minimal complexity into a scaffold. A biomimetic, nanofibrous hydrogel, co-assembled from two aromatic PAs, a structural unit, Fmoc-Phe-Phe-OH, and a functional unit, Fmoc-Arg-Gly-Asp-OH showed its supremacy to reconstruct a normal dermal tissue analog with enhanced mechanical strength. Fmoc-Phe-Phe-OH/Fmoc-Arg-Gly-Asp-OH hydrogel with elongated spindle-like morphology resembling myofibroblasts organized the nanofibrous 3D-networks and contracted the gel besides cell secretion of fibronectin and collagen I. As per *in vitro* results, fibronectin deposited ahead of denser collagen I, indicated construction of a normal dermal tissue within the scaffold. This simplified design by Zhou et al. (2014) supplies a pathway to create such versatile cell-scaffolds in skin engineering and similar bioactivities.

The policy to integrate *D*-amino acid residues indicates their potential use in drug delivery. The incorporation of *D*-amino acid residues in three chiral centers of homochiral tripeptide, Boc-Phe-Phe-Phe-COOH, could change the orientation of the corresponding aromatic ring of Phe residue(s) as well as the direction of π-π stacking interaction, which, in turn, could alter the mechanism of drug-delivery. The various combinations tried by Basu et al., hinted that the mechanical strength and proteolytic stability of a particular gelator could be modulated by changing the molecular chirality and placing the D-residue instead of L-residues in the appropriate position (at or toward the C terminus) (Basu et al., 2016).

A heterochiral supramolecular H-*D*-Leu-Phe-Phe-OH tripeptide hydrogel, loaded with an anti-metabolite and pyrimidine analog, hydrophobic antineoplastic drug, 5-fluorouracil (5-FU), formed hydrogel under physiological conditions and demonstrated a fast release. Interacting through π-π stacking between the H-Phe-Phe-OH and 5-FU rings as well as hydrogen bonding between the carboxylic acid group and N-H group of the 5-FU, this work examined the potential of a drug capable to interact non-covalently with a self-assembling tripeptide (Parisi et al., 2019). The outcome of the loading of the anti-inflammatory drugs, naproxen and ketoprofen, in the tripeptide hydrogel in earlier works could establish that the naphthalene unit engages more efficiently through non-covalent interactions than the benzene rings with the aromatic units of the peptide (Mayr et al., 2018). Though the release of 5-FU was found to be faster than that of naproxen, the role of π-π interaction and hydrogen bonding is concurrent with the hypothesized one. H-Phe-DAla-Phe-OH is an exception to the design rule, where the tripeptides of syndiotactic *L-D-L* stereochemistry adopt an amphipathic conformation, and the side chains in anisotactic configuration enabling the long-range self-organization turn into gel-forming fibers, as the molecule does not possess the required hydrophobicity and steric hindrance and so, it does not turn to hydrogel (Garcia et al., 2018). The attachment of *p*-aminobenzoyl moiety at the N-terminus of the tripeptide, (*p*-aminobenzoyl)- Phe-*D*-Ala- Phe-NH_2_, could address both the issues to yield a new gelator (Kieffer et al., 2019) that could be explored further.

The self-assembly of nanodrugs, as self-delivery of drugs, involving a photosensitizer (chlorine e6, Ce6) and Dox as carrier-free nanoparticles which combined photodynamic therapy with chemotherapy to inhibit tumor recurrence, has been tried (Zhang R. et al., 2016). Following the same working model, the supramolecular drug-drug delivery system (SDDDS) comprising of a tripeptide, H-Tyr-Ser-Val-OH (YSV) and Gefitinib (GEF), nanoparticles was studied. The low toxicity of the former and molecular targeting ability of the later led to the co-assembly, that required no chemical modification and performed better in drug efficacy both *in vitro* and *in vivo* as compared to individual drugs (Zhang Z. et al., 2017).

A phosphorylated and succinylated tripeptide, H-Gly-Tyr-Lys-OH, on conjugation with folic acid and Taxol, FA–Gly-Tyr-Lys–Taxol, self-assembled into a molecular hydrogel. The much higher weight percentage of Taxol in this gel compared to other similar gels, proposes it as injectable material to deliver Taxol for long period of time during chemotherapy (Wang and Yang, 2012).

A cytocompatible, photoresponsive hydrogelator with tetrazole-containing moiety, Tet(I)-Gly-Phe-Phe-OH, with fast responses to mild light irradiation, has shown the ability to modulate cellular microenvironment using a bio-orthogonal photoclick reaction. The mechanically strong hydrogel could entrap horse serum (HS) and human mesenchymal stem cells (hMSCs) for longer duration of time and its release could be modulated through photodegradation of the gel matrix (photo-triggered tetrazole-to-pyrazoline transformation). Further investigation holds bright chances to control the biological behavior of the live cells in spatially defined channels (He et al., 2013). Nap-Phe-Phe-Gly-OH, another tripeptide hydrogelator, was found to create surface-induced self-assembly that could inhibit human platelet aggregations. The comparison with Nap-Phe-Phe-OH, which could not inhibit the platelet aggregation, evidenced the status of Gly residue (Zheng et al., 2012). Further exploration on naphthaleneimide (NI) conjugates; NI-Gly-Phe-Phe-OH and NI-Phe-Phe-Gly-OH supramolecular hydrogels, articulated the good biocompatibility of NI-Gly-Phe-Phe-OH with cell lines. The observations could suggest that these supramolecular materials are promising candidates for their use in biomedical applications (Yeh et al., 2016).

Among the hydrogel biomaterials, a tripeptide, Ac-Phe-Phe-Ala-NH_2_, developed by Pospišil et al., self-assembled into non-covalent, transparent, stable and biocompatible nanofiber hydrogel scaffolds at physiological pH without use of any organic solvent. The intramolecular hydrogen bonding of cross-β-type structure aggregates and non-covalent interactions between molecules of hydrogelators were proposed to lead the creation of the 3D gel network. The physicochemical and biological properties (*in vitro*) of the hydrogel suggest it as a hopeful scaffold for tissue engineering applications (Pospišil et al., 2016).

Acknowledging the importance of various factors, viz., morphology, rigidity, periodicity and epitope spacing, which impacted the performance of nanostructures and in turn vividly sway cell adhesion and signaling, a peptide-based amphiphile with a rigid amphiphilic structure of the derivative was obtained. A novel compound, N-3β-(4-*tert*-butylbenzoylamine)-(7α,12α-dihydroxy-5β-cholan-24-oyl)-(*S*)-Arg-(*S*)-Gly-(*S*)-Asp (*tert*-but-PhC-RGD), containing the H-Arg-Gly-Asp-OH epitope, a recognition motif involved in cell adhesion processes, was synthesized. This unusual self-assembly, compared to conventional H-Arg-Gly-Asp-OH containing amphiphiles, formed fibers with a circular cross-section and tested positive as scaffolds for tissue regeneration. The circular dichorism results showed weak interactions among H-Arg-Gly-Asp-OH groups as compared to the usual twisted ribbons superstructure which stipulate specific interactions among the chiral groups of the amphiphiles. So, relatively free H-Arg-Gly-Asp-OH groups provided opportunities to interact with the environment and, thereby, were found vital to the applications (Travaglini et al., 2017).

A tri-β-peptide, Ac-βAla-Z-βLys-βAla-OH, self-assembled to form non-toxic, biocompatible and mechanically stable hydrogel, probably the first peptide hydrogel comprising exclusively of β-amino acids, with stiffness similar to brain tissue. This tripeptide contained a β-homo-lysine (K) residue (to enhance solubility of peptide in aqueous buffer) and a C14 acylated alkyl chain (a hydrophobic acyl tail to produce a peptide amphiphile to direct self-assembly into nano-cylinders) and formed stable hydrogels. The peptide monomers, driven by a 3-point hydrogen-bonding motif associated with the 14-helical structure of N-acetyl-β3–peptides, self-assembled into helices in a unique head to-tail fashion. The hydrogel could rapidly recover stiffness since high strain could break just the 3D fibrous network by upsetting the non-covalent interactions and transform to sol, without destroying the fibers, but reorganized to 3D network again on reducing the strain in a relatively short time. This superior self-healing property of the hydrogel with minimal post-operative damage could fasten the recovery (Del Borgo et al., 2013). Following the recipe, Motamed et al. (2016) had developed a new technique to synthesize peptide based self-assembly wherein helical N-acetyl-β3–peptides could spontaneously form fibers, ranging from nano- to macroscale. The β3-tripeptides were observed by Del Borgo et al. to devise distinct self-assembly units segregated by a linker and turn to fibrous assemblies. The linkers within the peptide sequence, whether rigid or flexible, could be composed of a bioactive α-peptide, could demonstrate fiber formation and create a hybrid α/β peptide scaffold regardless of amino acid sequence, or interruption of the sequence in the presented work (Del Borgo et al., 2018). A structural model developed for self-assembled N-acetyl-β3-peptides as stable three-stranded helical coiled coil could be used to explore the fibril structure for other materials (Christofferson et al., 2018) and be tailored for different applications (Kulkarni et al., 2019). A summary of this content is provided in Table 1.

**Table 1:** Summary of the amino acid/peptide derivatives and their applications (Figure 7).

**S.No**.	**Name of the amino acid/peptide**	**Derivative/co-assembly**		**Applications**	**References**
**Single amino acid**					
1	H-Trp-OH	Fmoc-Trp(Boc)-OH	Nanoparticles	Drug delivery; Dox, an anticancer drug	Dube et al., 2017
2	H-Lys-OH	Fmoc-Lys-OH	Nanoparticles	Drug delivery; Chlorin e6 (Ce6), a photosensitive drug	Liu et al., 2016
3	H-Phe-OH	Pyrene-Phe-OH	Hydrogel	Drug delivery; vit B_12_ and Dox	Nanda et al., 2013
4		H-*D*-Phe-OH	Flakes	Therapeutic molecule in phenylketonuria	Singh V. et al., 2014
5		Fmoc-Phe-OH	Hydrogel	Drug delivery; Dye as a model drug	Singh et al., 2015
6		Fmoc-F^5^-Phe-DAP	Hydrogel	Drug delivery; NSAID, diclofenac	Raymond et al., 2019
7	H-Cys-OH	N,N′-dibenzoyl-Cys-OH	Hydrogel	Drug delivery; Salicylic acid	Zhong et al., 2019
8	H-Trp-OH, H-Tyr-OH	-	Nanotubes	As drug carriers	Babar and Sarkar, 2017
9	H-Tyr-OH	H-Tyr(*tert*-Bu)-OH, H-*D*-Tyr(*tert*-Bu)-OH	Hydrogel	—-Do—	Aykent et al., 2019
10	H-Ala-OH	H-Ala-CAM, H-Ala-HYD, H-Ala- HYD+	Hydrogel	Drug delivery; Dox	Singh M. et al., 2014
11	H-Val-OH	H-3-methyl-2-(pyridin-4-ylmethylamino)-butanoic acid-OH/Zn^2+^	Hydrogel	Drug delivery; Caffeine as a model drug	Saha et al., 2014
12	H-Glu-OH	Co-assembly of C18–Glu-OH and analog; Click-Glu-OH (C18=N-stearoyl; Click= amide moiety in C18 replaced by 1,4-disubstituted 1,2,3-triazole unit)	Hydrogel	Drug delivery; antibiotic drug (vancomycin)	Bachl et al., 2015
13	H-His-OH	Fmoc-His-OH/Zn^2+^	Nanoparticles	Metallo-nanodrugs	Li S. et al., 2018
**Dipeptides**					
14	H-His-Phe-OH	Z-His-Phe-OH/Zn^2+^ (Z=N-benzyloxycarbonyl)	Nanoparticles	metallo-nanodrugs	Li S. et al., 2018
15	H-Phe-Phe-OH	H-Phe-Phe-OH	Microtubes	Drug delivery; Rhodamine B as model drug	Silva et al., 2013
16		H-Phe-Phe-OH/FA/Fe_3_O_4_	Nanotubes	Drug delivery; 5-FU, an antimetabolite drug and flufenamic acid, an anti-inflammatory cargo	Emtiazi et al., 2017
17		H-Phe-Phe-OH/FA	Nanotubes	Drug eluting stent	Zohrabi et al., 2016
18		H-Phe-Phe-OH	Nanotubes	Eradication of bacterial biofilms	Porter et al., 2018
19		TPP-Phe-Phe-OH	Nanodots	Photothermal therapy	Zou et al., 2017
20		Cationic H-Phe-Phe-OH/glutaraldehyde	Bola like nanocarriers	Drug delivery; Dox	Zhang H. et al., 2015
21		H-Phe-Phe-NH_2_·HCl/phosphotungstic acid	Nanofilms	Tissue engineering; photothermal treatment	Xing et al., 2015
22		H-Phe-Phe-ADA-Au	Nanospheres	Drug delivery; camptothecin	Li Q. et al., 2016
23		Nvoc-Phe-Phe-H	Hydrogel	Drug delivery; Insulin	Aldilla et al., 2018
24		H-His-Phe-OH/Hyaluronic acid	Hydrogel	Drug delivery (curcumin) and tissue engineering	Pujals et al., 2017
25		Fmoc-Phe-Phe-OH	Hydrogel	Drug delivery; Dox, 5-FU	Kuang and Xu, 2013
26		Fmoc-Phe-Phe-OH/Au	Organogel	Drug delivery; Dox	Roth-Konforti et al., 2018
27		Fmoc-Phe-Phe-OH	Hydrogel	Drug delivery; 5-FU, paclitaxel	Aviv et al., 2018
28		Fmoc-Phe-Phe-OH	Hydrogel	Biosensor, 3D scaffold	Erdogan et al., 2016
29		Fmoc-F^5^-Phe/Fmoc-Phe-Phe-OH	Hydrogel	Tissue engineering	Lian et al., 2017
30		Fmoc-Phe-Phe-OH/poly-L-lysine	Hydrogel	Drug delivery; Ce6	Lian et al., 2016
31		Fmoc-Phe-Phe-OH/Fmoc-Arg-OH/hydroxyapatite (HAP)	Hydrogel	Bone tissue regeneration	Xing et al., 2015
32		Boc-Phe-Phe-OH	Nanoparticles	Cancer therapy	Liyanage et al., 2015
33		H-Phe-Phe-OH Boc-Phe-Phe-OH	Fibers	Drug delivery/Tissue engineering	Creasey et al., 2016
34		Boc-β(O)-δ5-Phe-β(O)-δ5-Phe-OH	Hydrogel	Drug delivery; proflavine	Reja et al., 2019
35		rhein-Phe-Phe-OH	Nanospheres	Drug delivery; camptothecin	Sun et al., 2019
36		H-βPhe-Phe-OH/H-βPhe–ΔPhe-OH	Nanotubes	Drug delivery; anticancer drug, mitoxantrone	Parween et al., 2014
37				Drug delivery; cancer-testis antigens	Khatri et al., 2019
38	H-Arg-Phe-OH	H-Arg-ΔPhe-OH	Nanoparticles	Gene delivery	Khatri et al., 2017
39		H-Arg-ΔPhe-OH/Fe_3_O_4_	Nanoparticles	As site-specific delivery systems	Baskar et al., 2017
40		H-Arg-ΔPhe-OH	Nanoparticles	Drug delivery; Dox	Singh et al., 2018
41		H-Arg-ΔPhe-La	Nanoparticles	miR-199a-3p delivery	Varshney et al., 2018
42	H-Leu-Phe-OH	Amoc-Leu-Phe-OH	Hydrogel	In drug delivery and tissue engineering	Gavel et al., 2018b
43		H-Leu-ΔPhe-OH	Hydrogel	Drug delivery; mitoxantrone	Thota et al., 2016
44	H-Arg-Phe-OH/H-Lys-Phe-OH	H-Arg-ΔPhe-OH/H-Lys-ΔPhe-OH	Nanoparticles	Gene delivery	Panda et al., 2013
45	H-Trp-Phe-OH	Npx-Trp-*Z*-ΔPhe-OH	Hydrogel	Drug delivery: Npx	Vilaça et al., 2015a
46		H-Trp-Phe-OH/Zn^2+^	Nanoparticles	Drug delivery; Dox	Fan et al., 2016
47	H-Tyr-Phe-OH and H-Asp-Phe-OH	Npx-Tyr-*Z*-ΔPhe-OH/SPIONS and Npx-Asp-*Z*-ΔPhe-OH/SPIONS	Hydrogel	Drug delivery; Npx	Carvalho et al., 2019
48	H-Met-Phe-OH	Npx-Met-ZΔPhe-OH/manganese ferrite/gold nanoparticle and gold-decorated manganese ferrite nanoparticles	Hydrogel	Drug delivery; curcumin	Veloso et al., 2020
49	H-Lys-Lys-OH	Ac-Lys-Lys(CPT)-NH_2_ and NH_2_-Lys-Lys-(CPT)-NH_2_	Nanotubes 50	Protection of CPT from lactone hydrolysis	Kim et al., 2015
51		H-Lys-Lys-OH/5-FU	Nanotubes/Hydrogel	Drug delivery; 5-FU	Sun Y. et al., 2016
52		Fl-Leu-Leu-OH	β-sheet structure/nanotape	Fluorescent label	Kirkham et al., 2016
53	H-Asp-Phe-OH	H-Asp-Phe-OH	Nanoparticles	Drug delivery; calcitonin	Cao et al., 2017
54	H-Tyr-Phe-OH	NDI-Tyr-Phe-NH_2_	Nanofibers	Potential self-healer	Nalluri et al., 2014
55	H-glyoxylamide-Phe-OH	H-glyoxylamide-Phe-OH/various aromatic capping	Hydrogel	Drug delivery; ciprofloxacin	Aldilla et al., 2018
56	H-Phe-Arg-OH/H-Phe-Asp-OH	Fmoc-3F-Phe-Arg-NH_2_/Fmoc-3F-Phe-Asp-OH	Hydrogel	Tissue engineering	Liyanage et al., 2015
57	H-Glu—Glu-OH	N-octadecanoyl-1,5-bis(L-glutamic acid)-L-glutamic diamide/Mg^2+^	Nanofibers /Nanotubes /Nanobelts	Vit B_1_, anionic and cationic drugs (two-component drugs)	Qin et al., 2014
58	H-Trp-Tyr-OH	cyclo-*D*-Trp-Tyr	Nanotubes	CAP3 pRFP-C-RS DNA	Lee et al., 2015
59	H-Phe-Glu-OH	cyclo-Phe-Glu(O-*tert*-But)	Organogel /Hydrogel	Drug delivery; curcumin	Manchineella et al., 2017
60	Azo-Phe-Lys-OH	PAP-DKP-Lys	Hydrogel	DNA and doxorubicin delivery	Pianowski et al., 2016
**Tripeptides**					
61	H-Lys-Phe-Gly-OH	H-Lys-Phe-Gly-OH	Nanospheres (vesicles) /nanotubes	Drug delivery; Dox	Moitra et al., 2017
62	H-Leu-Leu-Leu-OH	Fmoc-Leu-Leu-Leu-OMe	Nanoparticles	Porphyrin derivative carriers, anticancer efficacy	Li J. et al., 2018
63	H-Pro-Phe-Gly-OH	Boc-Pro-Phe-Gly-OMe/Vit E-TPGS	Nanoparticles (vesicles)	Drug delivery; eosin, aspirin and curcumin	Yadav et al., 2015
64	H-Pro-Phe-Gly-OH	Boc-Pro-ΔPhe-Gly-OMe/Vitamin E-TPGS	Spherical nanostructures	Drug delivery; curcumin and ornidazole	Deka et al., 2017
65	H-AUDA-Phe-Phe-OH (AUDA=11-aminoundecanoic acid)	Boc-AUDA-Phe-Phe-OH	Hydrogel	Drug delivery; vancomycin and vit B_12_	Baral et al., 2014
66	H-Phe–Phe–Phe-OH	Fmoc-Phe–Phe–Phe-OH	Hydrogel	Drug delivery; dexamethasone	Chronopoulou et al., 2015
67	H-Phe-Phe-OH/H-Arg-Gly-Asp-OH	Fmoc-Phe-Phe-OH/Fmoc-Arg-Gly-Asp-OH	Hydrogel	Tissue engineering	Zhou et al., 2014
68	H-Phe-Phe-Phe-OH	Boc-Phe-Phe-Phe-OH, Various combinations of *D*-residue instead of *L*-residues	Hydrogel	Drug delivery; Dox	Basu et al., 2016
69	H-Leu-Phe-Phe-OH	H-*D*-Leu-Phe-Phe-OH	Hydrogel	Drug delivery;5-FU	Parisi et al., 2019
70	H-Tyr-Ser-Val-OH	H-Tyr-Ser-Val-OH/Gefitinib	Nanoparticles	Drug delivery; Drug mixture	Zhang Z. et al., 2017
71	H-Gly-Tyr-Lys-OH	FA–Gly-Tyr-Lys–Taxol	Hydrogel	Drug delivery; Taxol	Wang and Yang, 2012
72	H-Gly-Phe-Phe-OH	Tet(I)-Gly-Phe-Phe-OH	Hydrogel	Drug delivery; horse serum (HS) and human mesenchymal stem cells (hMSCs)	He et al., 2013
73	H-Phe-Phe-Gly-OH	Nap-Phe-Phe-Gly-OH	Hydrogel	Inhibition of human platelet aggregations	Zheng et al., 2012
74	H-Gly-Phe-Phe-OH	NI-Gly-Phe-Phe-OH	Hydrogel	For biomedical applications	Yeh et al., 2016
75	H-Phe-Phe-Ala-OH	Ac-Phe-Phe-Ala-NH_2_	Hydrogel	Suggested for tissue engineering	Pospišil et al., 2016
76	H-Arg-Gly-Asp-OH	N-3β-(4-t-butylbenzoyl-amine)-(7α,12α-dihydroxy-5β-cholan-24-oyl)-(*S*)-Arg-(*S*)-Gly-(*S*)-Asp (*tert*-but-PhC-RGD)	Hydrogel	Tissue engineering	Travaglini et al., 2017
77	H-Ala-Lys-Ala-OH	Ac-βAla-*Z*-βLys-βAla-OH	Hydrogel	Tissue engineering	Del Borgo et al., 2013

**Figure 7:**
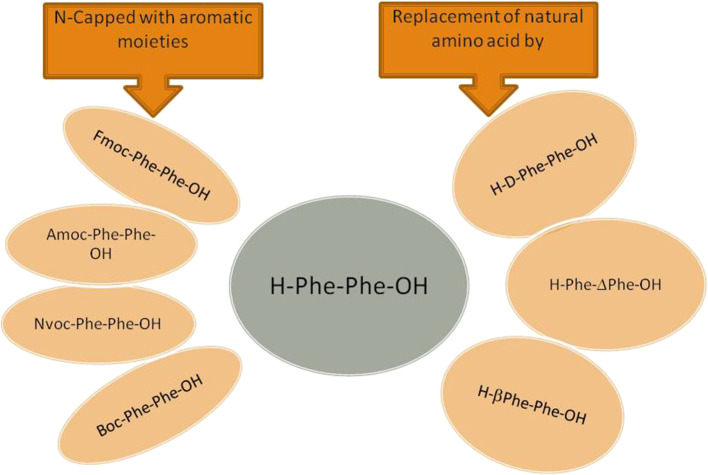
Some of the analogs of H-Phe-Phe-OH.

## Conclusion AND Future Perspectives

Self-assembly by bioactive molecules is the talent to associate into ordered 3D structures via noncovalent interactions, through a bottom-up approach, without guidance by an external source (Dehsorkhi et al., 2014). In the last 20 years, remarkable expansion has revolutionized, the aptitude has navigated to the potentially useful nanostructure-based materials and has offered newer tools in the field of biological and biomedical sciences. Nanotechnology, the driving force behind this evolution and the revolutionary modifications, has contributed immensely toward the realization of targeted and controlled delivery of available therapeutics. Different materials/systems have been developed with this vision. Except for few, these materials hold their own merits and demerits. Biocompatibility, the nature of the interaction with surrounding tissues, is crucial. Certain materials have claimed to demonstrate biocompatibility, while other developed materials showing toxicity, are proved unsuitable for *in vivo* applications. Cationic lipid-based nanostructures, found to activate immune system, are such an example (Campani et al., 2018). Besides, these are also linked with certain technological issues, such as, reproducibility, stability, low drug-loading, uncontrolled leaching and encapsulation problems. Further polymeric systems have been developed, but the evaluation studies showed these materials also facing similar limitations. The surface functionalization was considered to improve drug or gene-targeting which in general, is complicated (Abd Ellah and Abouelmagd, 2017). Likewise, natural polymers, elicited undesirable immune reactions, illustrated batch to batch inconsistency (Ginjupalli et al., 2017) and so *in vivo* performance of these polymers became debatable.

Peptides and small molecule-based nanostructures can be virtuous alternatives for therapeutic delivery due to characteristics, like, good biocompatibility, easy on design/synthesis and also the functionalization. These distinctive and superior qualities make their self-assembled nanostructures smart tools in biomedical field. The masterstroke lies in the ability of these self-assembled small molecules to exhibit stimuli-responsiveness to the environment (internal/external stimuli), thus enabled to control and sustain the therapeutic release as per the requirement, an exciting prospect for multiple biomedical and bionanotechnology applications in drug delivery (Mart et al., 2006). Thus, virtues like, mild and rapid synthesis setting, non-requirement of specialized equipments, low production expenditure, easy dispersibility in aqueous medium and simple functionalization promote their use as future candidates for diverse applications such as, production of biomaterials, healthcare systems drug/gene delivery, tissue engineering, imaging, sensors, diagnosis, bioelectronics, and so on. Diverse types of structures/architectures can be generated simply by altering the physical or chemical conditions. Thus, this newer area of research is growing at an accelerating pace. Self-assemblies do result in the a variety of type of structures; nanoparticles, nanospheres, nanotubes, nanorods, nanotapes, nanofibers, nanogels, etc., yet there still exist several challenges to be addressed to make these self- assemblies- the materials of choice for research. The limited information on the influence of nanostructures on the organisms, control over size and composition during processing, tenability, behavior in aqueous environment, stability, up-scaling and degree of loading/entrapment of therapeutics are some obstacles which still need sincere attention of the researchers. Besides, follow-on these studies to establish biocompatibility and immunogenicity of these nanostructures are lacking. Nonetheless, it is viable to fine-tune the physicochemical properties by assimilating chemical modifications and so optimize the peptide functionality to minimize the toxicity without threatening their therapeutic activity (Lombardi et al., 2019). Hydrogel based drug delivery systems are of special mention as these are customized for modified release to the target site, reduce toxicity and side effects (Du et al., 2015; Li Y. et al., 2016).

Peptides offer a striking platform as non-viral gene delivery vectors which can minimize the disadvantages associated with viral vectors. Several groups have deliberated on non-viral gene delivery vehicles coupled with targeting peptides (Levine et al., 2013; Sharma et al., 2014; Kang et al., 2019; Wang et al., 2019). Though several functional peptide classes possess essential characteristics to overcome extracellular and intracellular barriers and make gene delivery feasible, still none of these functional peptides have been observed to contain all the essential characteristics required to overcome all of the barriers. Attempts are being made to develop novel multifunctional peptide vectors by combinatorial strategies and high-throughput screening (Jia et al., 2013; Raad et al., 2014; Riley and Vermerris, 2017).

## Author Contributions

PK and AS conceived the idea to write a review article on peptide self-assembly. SG compiled the article and IS helped in the graphics and compilation. SG, AS, and PK also did editing.

## Conflict of Interest

The authors declare that the research was conducted in the absence of any commercial or financial relationships that could be construed as a potential conflict of interest.
